# Supplementary material for: Factors affecting the elderly’s behavioral intention toward smart home systems: A cross-sectional study from China’s eastern coast
Source: PLoS One. 2024 Dec 13;19(12):e0311280. doi: 10.1371/journal.pone.0311280 (PMC11643254; doi:10.1371/journal.pone.0311280)

PLOS ONE

Factors Affecting Elderly Behavioral Intention Towards Smart Home Systems: A Cross-Sectional Study from China's Eastern Coast

--Manuscript Draft--

**Manuscript Number:**	PONE-D-24-20607
**Article Type:**	Research Article
**Full Title:**	Factors Affecting Elderly Behavioral Intention Towards Smart Home Systems: A Cross-Sectional Study from China's Eastern Coast
**Short Title:**	Factors Affecting Elderly Behavioral Intention Towards Smart Home Systems
**Corresponding Author:**	Yuan Wang<br>Universiti Sains Malaysia<br>Penang, Pulau Pinang MALAYSIA
**Keywords:**	Smart Home Systems, Behavioral Intention, Factors, Elderly, Unified Theory of Acceptance and Use of Technology, Technology Acceptance
**Abstract:**	The increasing prevalence of aging populations alongside continuous advancements in smart technology underscores the pivotal role of smart home systems in elderly care. Despite this, studies have shown a low adoption rate of such systems among the elderly. Therefore, investigating the factors that influence the behavioral intention of elderly people towards smart home systems is imperative. This study aims to address this gap by constructing a theoretical model of elderly user behavioral intention, drawing upon the Technology Acceptance Model (TAM) and the Unified Theory of Acceptance and Use of Technology (UTAUT). Employing empirical analysis via Partial Least Squares Structural Equation Modeling (PLS-SEM), this research elucidates the mechanisms through which various factors impact behavioral intention. The findings reveal that facilitating conditions emerge as the primary positive direct factor influencing the behavioral intention of smart home systems among the elderly, thereby confirming its central role in the UTAUT framework. Conversely, perceived cost emerges as a significant negative direct influencing factor, underscoring the importance of pricing considerations in the elderly's adoption of smart home systems. Moreover, compatibility, perceived usefulness, and perceived ease of use exhibit positive direct effects on behavioral intention. It is noteworthy that compatibility and facilitating conditions exert significant indirect positive effects on behavioral intention through perceived ease of use, while perceived trust influences behavioral intention indirectly through perceived usefulness. Furthermore, perceived usefulness is influenced by social influence, perceived ease of use, compatibility, and facilitating conditions, highlighting its pivotal mediating role in the model. While the direct impact of perceived trust on behavioral intention remains unconfirmed, its significant indirect impact is established. This study validates the effectiveness of TAM and UTAUT frameworks in the context of smart home systems for the elderly in China's eastern coastal regions, thereby contributing theoretically by confirming their applicability in this specific context. The practical implications of this research extend to elderly people, smart home system developers and designers, as well as government and relevant agencies.
**Order of Authors:**	Yuan Wang
	Norazmawati Md. Sani
	Yinhong Hua
	Qianling Jiang
	Long Zhao
**Additional Information:**	
**Question**	**Response**
**Financial Disclosure**	The author(s) received no specific funding for this work.

Powered by Editorial Manager® and ProduXion Manager® from Aries Systems Corporation

Enter a financial disclosure statement that describes the sources of funding for the work included in this submission. Review the submission guidelines for detailed requirements. View published research articles from *PLOS ONE* for specific examples.<br><br>This statement is required for submission and **will appear in the published article** if the submission is accepted. Please make sure it is accurate.<br><br>**Funded studies**<br>Enter a statement with the following details:<br>• Initials of the authors who received each award<br>• Grant numbers awarded to each author<br>• The full name of each funder<br>• URL of each funder website<br>• Did the sponsors or funders play any role in the study design, data collection and analysis, decision to publish, or preparation of the manuscript?<br><br>Did you receive funding for this work?	
**Competing Interests**<br><br>Use the instructions below to enter a competing interest statement for this submission. On behalf of all authors, disclose any competing interests that could be perceived to bias this work—acknowledging all financial support and any other relevant financial or non-financial competing interests.<br><br>This statement is required for submission and **will appear in the published article** if the submission is accepted. Please make sure it is accurate and that any funding sources listed in your Funding Information later in the submission form are also declared in your Financial Disclosure statement.<br><br>View published research articles from	The authors have declared that no competing interests exist.

PLOS ONE for specific examples.<br><br>**NO authors have competing interests**<br><br>Enter: *The authors have declared that no competing interests exist.*<br><br>**Authors with competing interests**<br><br>Enter competing interest details beginning with this statement:<br><br>*I have read the journal's policy and the authors of this manuscript have the following competing interests: [insert competing interests here]*<br><br>* typeset	
**Ethics Statement**<br><br>Enter an ethics statement for this submission. This statement is required if the study involved:<br><br>• Human participants<br>• Human specimens or tissue<br>• Vertebrate animals or cephalopods<br>• Vertebrate embryos or tissues<br>• Field research<br><br>Write "N/A" if the submission does not require an ethics statement.<br><br>General guidance is provided below. Consult the submission guidelines for detailed instructions. **Make sure that all information entered here is included in the Methods section of the manuscript.**	This study was reviewed and approved by Qufu Normal University Biomedical Ethics Committee, with the approval number: 2024051. All respondents provided written informed consent to participate in the study.

Powered by Editorial Manager® and ProduXion Manager® from Aries Systems Corporation

**Format for specific study types**<br><br>**Human Subject Research (involving human participants and/or tissue)**<br>• Give the name of the institutional review board or ethics committee that approved the study<br>• Include the approval number and/or a statement indicating approval of this research<br>• Indicate the form of consent obtained (written/oral) or the reason that consent was not obtained (e.g. the data were analyzed anonymously)<br><br>**Animal Research (involving vertebrate animals, embryos or tissues)**<br>• Provide the name of the Institutional Animal Care and Use Committee (IACUC) or other relevant ethics board that reviewed the study protocol, and indicate whether they approved this research or granted a formal waiver of ethical approval<br>• Include an approval number if one was obtained<br>• If the study involved *non-human primates*, add *additional details* about animal welfare and steps taken to ameliorate suffering<br>• If anesthesia, euthanasia, or any kind of animal sacrifice is part of the study, include briefly which substances and/or methods were applied<br><br>**Field Research**<br><br>Include the following details if this study involves the collection of plant, animal, or other materials from a natural setting:<br>• Field permit number<br>• Name of the institution or relevant body that granted permission	
**Data Availability**<br><br>Authors are required to make all data underlying the findings described fully available, without restriction, and from the time of publication. PLOS allows rare exceptions to address legal and ethical concerns. See the PLOS Data Policy and FAQ for detailed information.	No - some restrictions will apply

Powered by Editorial Manager® and ProduXion Manager® from Aries Systems Corporation

A Data Availability Statement describing where the data can be found is required at submission. Your answers to this question constitute the Data Availability Statement and **will be published in the article**, if accepted.<br><br>**Important:** Stating 'data available on request from the author' is not sufficient. If your data are only available upon request, select 'No' for the first question and explain your exceptional situation in the text box.<br><br>Do the authors confirm that all data underlying the findings described in their manuscript are fully available without restriction?	
**Describe where the data may be found in full sentences. If you are copying our sample text, replace any instances of XXX with the appropriate details.**<br><br>• If the data are **held or will be held in a public repository**, include URLs, accession numbers or DOIs. If this information will only be available after acceptance, indicate this by ticking the box below. For example: *All XXX files are available from the XXX database (accession number(s) XXX, XXX.*).<br>• If the data are all contained **within the manuscript and/or Supporting Information files**, enter the following: *All relevant data are within the manuscript and its Supporting Information files.*<br>• If neither of these applies but you are able to provide **details of access elsewhere**, with or without limitations, please do so. For example:<br><br>*Data cannot be shared publicly because of [XXX]. Data are available from the XXX Institutional Data Access / Ethics Committee (contact via XXX) for researchers who meet the criteria for access to confidential data.*<br><br>*The data underlying the results presented in the study are available from (include the name of the third party*	To protect the privacy of the respondents, the data supporting the findings of this study are available upon request from the corresponding author.

and contact information or URL).<br>• This text is appropriate if the data are owned by a third party and authors do not have permission to share the data.<br><br>* typeset	
Additional data availability information:	Tick here if the URLs/accession numbers/DOIs will be available only after acceptance of the manuscript for publication so that we can ensure their inclusion before publication.

Powered by Editorial Manager® and ProduXion Manager® from Aries Systems Corporation

manuscript Click here to access/download;Manuscript;Manuscript.docx

Factors Affecting Elderly Behavioral Intention Towards Smart Home Systems: A Cross-Sectional Study from China's Eastern Coast

Yuan Wang [1*], Norazmawati Md. Sani [2], Yinhong Hua[3], Qianling Jiang[4], Long Zhao[5]
[1] School of Housing, Building and Planning, Universiti Sains Malaysia, Penang, Malaysia
[2] School of Housing, Building and Planning, Universiti Sains Malaysia, Penang, Malaysia
[3] School of Art Design, Qufu Normal University, Rizhao, China
[4] School of Design, Jiangnan University, Wuxi, China
[5] School of Art Design, Qufu Normal University, Rizhao, China
[*]Corresponding Author: yuanwang@student.usm.my

Abstract: The increasing prevalence of aging populations alongside continuous advancements in smart technology underscores the pivotal role of smart home systems in elderly care. Despite this, studies have shown a low adoption rate of such systems among the elderly. Therefore, investigating the factors that influence the behavioral intention of elderly people towards smart home systems is imperative. This study aims to address this gap by constructing a theoretical model of elderly user behavioral intention, drawing upon the Technology Acceptance Model (TAM) and the Unified Theory of Acceptance and Use of Technology (UTAUT). Employing empirical analysis via Partial Least Squares Structural Equation Modeling (PLS-SEM), this research elucidates the mechanisms through which various factors impact behavioral intention. The findings reveal that facilitating conditions emerge as the primary positive direct factor influencing the behavioral intention of smart home systems among the elderly, thereby confirming its central role in the UTAUT framework. Conversely, perceived cost emerges as a significant negative direct influencing factor, underscoring the importance of pricing considerations in the elderly's adoption of smart home systems. Moreover, compatibility, perceived usefulness, and perceived ease of use exhibit positive direct effects on behavioral intention. It is noteworthy that compatibility and facilitating conditions exert significant indirect positive effects on behavioral intention through perceived ease of use, while perceived trust influences behavioral intention indirectly through perceived usefulness. Furthermore, perceived usefulness is influenced by social influence, perceived ease of use, compatibility, and facilitating conditions, highlighting its pivotal mediating role in the model. While the direct impact of perceived trust on behavioral intention remains unconfirmed, its significant indirect impact is established. This study validates the effectiveness of TAM and UTAUT frameworks in the context of smart home systems for the elderly in China's eastern coastal regions, thereby contributing theoretically by confirming their applicability in this specific context. The practical implications of this research extend to elderly people, smart home system developers and designers, as well as government and relevant agencies.

Keywords: Smart Home Systems, Behavioral Intention, Factors, Elderly, Unified Theory of Acceptance and Use of Technology, Technology Acceptance

1. Introduction

Most countries around the world have experienced or are on the brink of experiencing population booms. Coupled with declining birth rates and mortality rates, the global elderly population is evidently poised to reach unprecedented levels [1]. In China, the situation is no exception. According to the National Bureau of Statistics and the 'China Development Report 2020,' in 2025, the elderly population aged 65 and above is projected to exceed 210 million, constituting approximately 15% of the total population. By 2035 and 2050, this demographic is expected to reach 310 million and nearly 380 million, respectively, representing 22.3% and 27.9% of China's total population [2]. In terms of regional demographics, the aging population is most pronounced along the eastern coastal areas, such as Shandong, Jiangsu, Guangdong, Hebei, Zhejiang, Liaoning, Guangxi, Fujian, Shanghai and Hainan [3].

From the perspective of a nation and society, widespread aging leads to increased family expenditures, strains on public finances and healthcare systems, as well as slowing economic growth [4-6]. Meanwhile, from the perspective of the elderly themselves, advancing age presents challenges such as physical decline and health issues [7], along with feelings of loneliness [8, 9]. Therefore, aging at home [10] and maintaining independent living [11] have become imperative for the elderly in their later years.

With the continuous advancements in smart technology, research confirms that smart homes play a significant role in enhancing independence, quality of life, and overall well-being for the elderly [12-16]. Empowered by technologies like the Internet of Things, information technology, big data, and cloud computing, smart homes have advanced significantly. They can record, store, and analyze users' habits and needs [17], interact through sound, light, location, and images [18], and connect with external resources to offer diverse services, including health, assistance, security, education, and entertainment [19]. AI-powered smart technologies and environments are swiftly evolving to aid overwhelmed caregivers, facilitating their access to medical services, and mitigating isolation and depression in the elderly [20].

While smart homes play an essential role in supporting the independent living and well-being of the elderly in their later years [13-16], existing studies indicate a lower adoption rate among the elderly regarding smart homes [21-23]. Therefore, it is necessary to explore which factors influence the behavioral intention of smart home systems among the elderly.

2. Literature Review

Based on a review of articles concerning smart homes for the elderly over the past decade (2014-2023), the findings indicate that research on international smart homes for the elderly primarily focused on various aspects of smart home technologies [24-26]. For example, Li et al. [24] investigated health monitoring using wearable technologies for the elderly, Arthanat et al. [25] identified determinants of information communication and acceptance of smart home automation technology for aging-in-place, and Wei et al. [26] studied the willingness of community elderly to adopt smart

home technology, developing an extended technology acceptance model.

From the perspective of China, research on smart homes for the elderly predominantly centers on aspects such as devices, interface, technology, service, and care [27-31]. For example, Chiu et al. [27] explored the acceptance of robots and pets as life partners in later life. some scholars have proposed smart home user interface design strategies and practices tailored to meet the needs of elderly people [32]. Forestal and Li [31] systematically reviewed prior research on the willingness of elderly people to embrace smart home technologies. Furthermore, researchers explored the application and prospects of intelligent service robots in assisting Chinese elderly people to age in place.

From a theoretical model perspective, previous research on smart homes for the elderly has mainly been based on TAM and UTAUT (presented in Table 1). In terms of influencing factors, prior relevant studies have confirmed that the factors with strong explanatory power, ranked from high to low, are: perceived usefulness (PU), perceived ease of use (PEOU), social influence (SI), facilitating conditions (FC), perceived cost (PC), attitude towards use (AT), perceived trust (PT), performance expectancy (PE) and compatibility (COM) (presented in Table 2).

Table 1. Theories and Models of Smart Homes for the Elderly Using Quantitative Methods Over the Past 10 Years (2014-2023)

Authors and Years	Theories and models
Chen & Chan, 2014	UTAUT & TAM
Ma et al., 2016	UTAUT & TAM
Pal et al., 2018	UTAUT & TAM
Pal et al., 2018	TAM
Li et al., 2019	UTAUT & TAM
Etemad-Sajadi & Gomes Dos Santos, 2019	TAM
Arar et al., 2021	UTAUT
Maswadi et al., 2022	UTAUT
Zhong et al., 2022	UTAUT
Yan & Lee, 2022	TAM
Wei et al., 2023	TAM

Table 2. Factors that affect the Smart Homes for the Elderly Using Quantitative Method Over the Past 10 Years (2014-2023)

Factors	Sources	Frequency
Perceived Usefulness / Usefulness	[24, 26, 28, 33-39]	10

Perceived Ease of Use / Ease of Use	[26, 28, 33-39]	9
Social Influence/ Subjective Norm	[25, 34, 35, 39-42]	7
Facilitating Conditions	[24, 30, 33, 39, 40, 42]	6
Perceived Cost /Cost Tolerance	[33, 35, 43-45]	5
Attitude (towards Use)	[28, 33-35, 42]	5
Perceived Trust / Trust	[36-38, 43]	4
Performance Expectancy / Expected Performance	[30, 40, 42, 43]	4
Compatibility /Interoperability	[24, 30, 38, 44]	4

Although previous studies have extensively researched smart homes for the elderly, research on factors influencing the behavioral intention of smart home systems by the elderly remains limited. Furthermore, there is currently no research specifically targeting the heavily aged population in the eastern coastal regions of China. Therefore, this study establishes a comprehensive model for smart home systems for the elderly based on TAM and UTAUT theories and models, aiming to validate its applicability in different regions and cultural backgrounds.

The research objectives of this study are:

1） To identify the factors affecting smart home systems for the elderly in eastern coastal areas of China.

2） To examine the relationship between the factors affecting smart home systems for the elderly in eastern coastal areas of China.

3） To evaluate the applicability of TAM and UTAUT in smart home systems among the elderly in eastern coastal areas of China.

3. Theoretical Framework and Research Hypotheses

3.1 Theoretical Framework

The literature review confirms the effectiveness of TAM and UTAUT theories in the context of smart homes. Yet, research on these theories for elderly-centric smart home systems remains scarce. Therefore, this study aims to fill this gap by developing a comprehensive model based on TAM and UTAUT to explore factors shaping elderly people' behavioral intention towards smart home systems.

3.1.1 Technology Acceptance Model (TAM)

Proposed by Davis in 1989, the TAM has emerged as the predominant theoretical framework [46]. In its initial version, TAM comprised four constructs: the behavioral intention (BI), attitude (ATT), perceived ease of use (PEOU) and perceived usefulness (PU). BI is influenced by both ATT and PU, with ATT being shaped by PU and PEOU. Moreover, there is a notable correlation between PEOU and PU [35]. However, recent research, such as the STAM model and studies related to smartphones among the elderly [33, 39], have indicated that ATT does not significantly impact BI. Therefore, this construct is not considered in the current context.

TAM has been extensively employed as a theoretical framework for exploring the intention to use diverse information communication technologies and intelligent systems. Its robustness in forecasting the inclination towards adopting smart technologies or services has been firmly established [35]. Recent studies corroborate this theoretical framework. Pal et al. [35] examined the determinants of the elderly's adoption of smart home services, utilizing TAM. Etemad-Sajadi and Gomes Dos Santos [36] scrutinized the acceptance of connected health technologies among senior citizens in their residences employing TAM. Yan & Lee [38] delved into and formulated a model for a smart home healthcare system for the elderly, grounded in TAM. Wei et al. [26] investigated the readiness of elderly community members to embrace smart home technologies, drawing upon TAM. Together, these studies underscore TAM's efficacy in elucidating behavioral intention. Moreover, findings from literature reviews indicate that PU and PEOU exhibit robust explanatory power in the context of smart homes for the elderly. Consequently, this study opts to employ PU and PEOU as research variables.

● Perceived Usefulness (PU)

PU is defined subjectively as users' belief that a particular system will improve their performance within an organizational setting [46]. In the context of this study, PU refers to the perceived benefits that the elderly associate with smart home systems.

● Perceived Ease of Use (PEOU)

PEOU is characterized as the extent to which an individual perceives that utilizing a specific system will demand minimal effort [46]. In the scope of this study, PEOU denotes the anticipated effort the elderly expects in embracing smart home systems.

3.1.2 Unified Theory of Acceptance and Use of Technology (UTAUT)

The UTAUT draws upon a synthesis of eight theories: the Theory of Planned Behavior (TPB), the Technology Acceptance Model(TAM), the combination of TAM and TPB, the Theory of Reasoned Action, the Model of Personal Computer Utilization, the Diffusion of Innovation Theory, Social Cognitive Theory and the Motivation Model [47]. The model encompasses four constructs: social influence (SI), and facilitating conditions (FC), performance expectancy (PE) and effort expectancy (EE).

UTAUT demonstrates its efficacy in the realm of smart homes, as demonstrated by multiple investigations. Arar et al. [40] analyzed the acceptance and preferences of smart home technology among the elderly in Dubai, UAE, employing the UTAUT framework. Maswadi et al. [42] investigated the determinants affecting the behavioral intention of the elderly to adopt smart home technologies in Saudi Arabia utilizing the UTAUT model. Furthermore, Zhong et al. [30] conducted a comparative study on the acceptance variances of smart home voice assistants among younger, middle-aged, and the elderly, employing the UTAUT framework.

A comparison between TAM and UTAUT reveals that the PU in TAM corresponds to the PE in the UTAUT model [42, 48]. Given that PU has been selected as a research construct in TAM for this study, PE is therefore not chosen further. Furthermore, through literature review, it is evident that Social Influence (SI) and Facilitating Conditions (FC) exhibit strong explanatory power in the context of smart homes for the elderly. Hence, this study selects SI and FC as research constructs.

● Social Influence (SI)

SI encompasses alterations in an individual's perceptions, attitudes, or behaviors resulting from the impact of individuals or groups [47]. Previous research indicates that family, friends, governmental entities, and mass media play a constructive role in fostering the acceptance of smart homes among the elderly [39, 49-51]. In the scope of this study, SI refers to the perceptions and attitudes of the elderly affected by families, friends, government and mass media.

● Facilitating Conditions (FC)

FC is defined as perceptions of factors in an environment that support the use of a particular technology [47, 52]. Previous studies have suggested that FC encompasses variables such as an individual’s educational background and the availability of financial and technical assistance from various sources [39, 47, 53]. In the scope of this research, FC relates to the perceived factors that bolster the behavioral intention of the elderly towards smart home systems.

Furthermore, literature reviews demonstrate that in studies related to smart homes for the elderly based on TAM and UTAUT, many researchers have established extended models that confirm the significance of Perceived Trust (PT) [36-38, 43]，Perceived Cost (PC) [33, 35, 43-45] and Compatibility (COM) [24, 30, 38, 44] in smart homes

for the elderly. Hence, this study also selects PT, PC, and COM as research constructs.

● Perceived Trust (PT)
PT is characterized as the psychological state in which the elderly believe that their personal data will be secure, carefully guarded, and kept anonymous [43]. Within the framework of this study, PT denotes the mindset of the elderly regarding the safety, protection, and anonymity associated with utilizing smart home systems.

● Perceived Cost (PC)
PC is described as the monetary value that elderly users deem suitable to sacrifice in exchange for the services provided by utilizing smart homes [43]. Within the scope of this study, PC denotes the economic worth that the elderly perceives as an acceptable trade-off for the benefits obtained from using smart home systems.

● Compatibility (COM)
COM is defined as the extent to which a technology aligns with the technical features of other prevailing products and with the requirements and lifestyle choices of users [24, 54]. In the scope of this study, COM refers to the degree to which a smart home system corresponds to the technical capabilities of existing products, such as smartphones and tablets, and fulfills the needs and lifestyle preferences of the elderly.

3.2 Research Hypotheses and Model Construction

Previous research, which investigates the acceptance of smart home technology among the elderly, consistently emphasizes the relationship among PU, PEOU, and BI [26, 35, 37]. Specifically, Pal et al. [35] demonstrated that PEOU positively influenced PU, while both PU and PEOU had positive effects on BI in the context of smart home services. Furthermore, Song et al. [37] and Wei et al. [26] corroborated these findings, confirming the significant positive influence of PEOU on PU and the considerable impact of PEOU on BI. Consequently, the following hypotheses are posited:
H1: PEOU significantly and positively affects PU.
H2: PU significantly and positively affects BI.
H3: PEOU significantly and positively affects BI.

The impact of FC on PEOU, PU, and BI, as one of the variables in UTAUT, has been substantiated in several studies. Chen and Chan [39] validated that FC significantly influences PEOU, PU, and BI in the senior technology acceptance model. Similarly, Li et al. [24] showcased that FC positively influences both PEOU and BI in the smart wearable acceptance model. Given that smart home systems frequently utilize community and wireless networks to convey ubiquitous health monitoring data, FC assumes particular importance in predicting BI. Consequently, the following hypotheses are posited:
H4: FC significantly and positively affects PU.
H5: FC significantly and positively affects PEOU.
H6: FC significantly and positively affects BI.

Previous studies have validated the correlation between SI and PU. Influential individuals such as family and friends often sway individuals' emotions, opinions, and behaviors [24]. In the research titled'Health monitoring through wearable technologies for elderly people: Smart wearables acceptance model,' Li et al. [24] affirmed the favorable influence of SI on PU. In the context of smart home systems, SI measures the influence of important family members, companion and commercial advertising. Consequently, the following hypothesis is posited:
H7: SI significantly and positively affects PU.

Previous study has indeed confirmed that trust in technology significantly and positively influences PU [55]. Research has indicated that an augmentation in PT enhances the Performance Expectancy of elderly users' acceptance of smart homes for healthcare purposes [43]. Furthermore, as previous researchers have proposed the equivalence of PU in TAM to Performance Expectancy in UTAUT [42], it follows that PT positively influences the PU embraced by smart homes for the elderly. Additionally, prior studies have demonstrated that an augmentation in PT positively impacts the BI of elderly users in accepting smart homes for healthcare purposes [43]. Consequently, the following hypotheses are posited:
H8: PT significantly and positively affects PU.
H9: PT significantly and positively affects BI.

The cost of any new technology, as determined by PC, plays a pivotal role in its success [55, 56]. Elevated costs associated with smart home systems may adversely affect the mindset of the elderly. Clearly, if the cost is deemed unreasonable, users are likely to exhibit resistance in adopting these products or services [43, 57]. Pal et al. [43] posited and substantiated that PC exerted a negative influence on BI regarding smart homes for elderly healthcare. Consequently, the following hypothesis is posited:
H10: PC significantly and negatively affects BI.

Compatibility (COM) holds significant importance in influencing the acceptance of smart wearable technologies among the elderly. In the context of smart home systems, it is imperative to assess technical compatibility with current devices like smartphones and wireless sensor networks to gauge the ability to transmit monitoring information to remote devices. Previous research has affirmed the substantial impact of COM on PEOU, PU, and BI [24, 35]. Consequently, the following hypotheses are posited:
H11: COM significantly and positively affects PU.
H12: COM significantly and positively affects PEOU.
H13: COM significantly and positively affects BI.

3.3 Model Construction

The research model depicted in Figure 1 is formulated in accordance with the hypotheses mentioned earlier. This model integrates five independent variables: Social Influence (SI), Facilitating Conditions (FC), Perceived Trust (PT), Perceived Cost (PC),

and Compatibility (COM). Additionally, it encompasses two mediating variables: Perceived Usefulness (PU) and Perceived Ease of Use (PEOU). Moreover, it comprises one dependent variable: Behavioral Intention (BI).

Figure 1. Research Modeling of Structural Equation. Note：

Red – Social Influence (SI) and Facilitating Conditions (FC) are fundamental constructs in the UTAUT model, serving as independent variables. Perceived Trust (PT), Compatibility (COM), and Perceived Cost (PC) are constructs substantiated in pertinent research drawing from TAM and UTAUT, showcasing notable influence as independent variables.

Yellow – Perceived Usefulness (PU) and Perceived Ease of Use (PEOU) are fundamental constructs in the TAM model, serving as mediating variables.

Blue –Behavioral Intention, derived from both TAM and UTAUT, serving as the dependent variable.

Each arrow within the model denotes a hypothesis (H1 to H13), illustrating the directional influence from one construct to another. This extensive model is designed to encompass the diverse factors that impact the behavioral intention of the elderly towards smart home systems, furnishing a systematic framework for analyzing their behavioral intention.

4. Research Design and Methodology

4.1 Questionnaire Design

The measurement scale utilized in this study was carefully designed and refined, drawing insights from relevant research. The questionnaire comprises two primary sections: demographic information and measurement items. While the first section is informed by prior studies, the second section is derived from the measurement items of the scale. Subsequently to the initial drafting of the questionnaire, researchers conducted a pilot test involving 35 participants and solicited feedback from three domain experts. Incorporating their suggestions, researchers enhanced the questionnaire by providing detailed explanations of pertinent concepts and making linguistic adjustments to ensure its accuracy and comprehensibility. Table 3 delineates the definitive variable definitions alongside their respective sources.

Table 3． Measurement items.

Construct	Item	Question (responses based on 7 points Likert scale)	References
Perceived Usefulness	PU 1	I will accomplish my daily activities more quickly by using smart home systems.	[58-60]
	PU 2	Using smart home systems will enhance my overall quality of life.	

	PU 3	Using smart home systems will make my life more convenient.	
	PU 4	Using smart home systems will make my life useful.	
	PU 5	Using smart home systems will make my life more enjoyable.	
Perceived Ease of Use	PEOU 1	I will operate smart home systems in my home by myself.	[33, 35]
	PEOU 2	I will find smart home systems easy to use.	
	PEOU 3	Using smart home systems will not require any special mental or physical effort.	
	PEOU 4	It will be easy and clear for me to use the smart home system presented in my home.	
	PEOU 5	I will be skillful at using smart home systems.	
Social Influence	SI 1	I will use smart home systems if media/government encourages to use them.	[34, 43]
	SI 2	I will use smart home systems in my house if my family members and friends do so.	
	SI 3	I will use smart home systems if people whose opinion I value recommend me to do so.	
	SI 4	People who are important to me will support my use of smart home systems.	
Facilitating Conditions	FC 1	It is important to have someone who can help me tackle the problems in the use of smart home systems.	[33, 43]
	FC 2	Training practice is useful and important for the use of smart home systems.	
	FC 3	I believe proper guidance will be available when using smart home systems.	
	FC 4	I believe proper service is available if I face difficulty in using smart home systems.	
Perceived Trust	PT 1	I fear to use smart home systems due to loss of my personal data and privacy.	[43]
	PT 2	The internet offers a medium through which sensitive personal information can	

		be sent confidentially.	
	PT 3	I think it risky to disclose my personal details and health information to the smart home service providers.	
Perceived Cost	PC 1	The daily cost of smart home systems should be economical.	[33, 43]
	PC 2	I will need to pay a much lower price for traditional home devices than subscribing to smart home systems.	
	PC 3	Purchasing and maintaining smart home systems will be a burden for me.	
	PC 4	The cost of investing smart home systems is too expensive.	
Compatibility	COM 1	Smart home systems can be compatible with my existing electronics (smartphone and others).	[24, 35]
	COM 2	Purchasing smart home devices from different vendors will not create any operational problem.	
	COM 3	The smart home devices can inter-operate with each other.	
	COM 4	Using smart home systems will fit into all aspects of my work.	
	COM 5	Using smart home systems will not affect my daily life.	
Behavioral Intention	BI 1	I will be interested in smart home systems.	[24, 34, 43]
	BI 2	Using smart home systems is a good idea.	
	BI 3	I expect to use smart home systems in my house.	
	BI 4	I will definitely use smart home systems in the near future.	
	BI 5	I intend to invest and use smart home systems as much as possible.	

4.2 Sample and Data Collection

4.2.1 Respondents

Respondents were elderly people aged 55 years and above from the eastern coastal regions of China. The selection of this age criterion was based on relevant studies conducted over the past decade [33, 39, 43, 44]. Furthermore, according to prior research, respondents are required to have a certain level of understanding or experience in smart home or system usage [38]. Respondents were briefed on the objectives and intentions of the study, as well as the overarching objectives of the research team.

4.2.2 Sample Size

The sample size was determined using a formula. This study utilized Cochran's sample size formula, which is employed to ascertain an optimal sample size for a desired level of precision and is recommended for studies with large populations (such as 10-15K) or infinite populations [61].

The formula used for sample size calculation is as follows:

$$n_{55} = \frac{z^2. \ \ p \ .(1-p)}{e^2}$$

n_{55}: sample size of individuals aged 55 years and above.
e: desired level of precision, representing the margin of error.
p: the proportion of the population (expressed as a percentage) exhibiting the characteristic.
z: the z-value, obtained from a standard normal distribution table.

Substituting the given values into the formula yields:

$$n_{55} = \frac{1.96^2. 0.5 \ .(1-0.5)}{0.05^2} = 384.16 \approx 385$$

Consequently, a minimum sample size of 385 is necessary for this study. Previous research suggested that achieving a response rate of 60% is commendable, whereas reaching 70% indicates a significantly high response level [62]. To enhance the response rate, this study aims to achieve a 70% response rate.

$$\frac{385}{n} = 70\%$$

$$\mathrm{n} = 550$$

Therefore, the inclusion of 550 samples is essential to ensure the precision of the research outcomes.

4.2.3 Sampling procedure

This study employed a stratified sampling approach to enlist a demographically representative cohort of elderly people in China, specifically targeting regions along the eastern coast where aging is notably pronounced. To mitigate potential errors, it was imperative to precisely delineate the attributes and criteria defining the study respondents during the sampling process. To address the risk of sample bias, the sampling distribution was established in accordance with the respective elderly population proportions across each eastern coastal province, thereby constraining participant numbers from varying provinces. The outcomes are delineated in Table 4.

Table 4. Sample size of elderly people by region

Region	Number of elderly people	Proportion of elderly population (%)	sample size
Shandong	21220806	18.4	102
Jiangsu	18505345	16	89
Guangdong	15565066	13.5	75
Hebei	14812048	12.8	65
Zhejiang	12072700	10.5	58
Liaoning	10954467	9.5	53
Guangxi	8363779	7.2	40
Fujian	6637869	5.8	32
Shanghai	5815462	5.0	28
Hainan	1476599	1.3	8

4.2.4 Ethical considerations

This study underwent review and approval by the Qufu Normal University Biomedical Ethics Committee and was conducted in accordance with relevant guidelines/ regulations. Before completing the questionnaire, written informed consent was obtained from all respondents.

4.2.5 Data collection

The recruitment period for this study was from March 29, 2024 to April 12, 2024. The questionnaires were distributed through the online survey platform (Questionnaire Star website). To ensure respondents possessed a fundamental understanding of smart home systems, researchers provided explanations regarding basic concepts and descriptions of such systems during the recruitment phase. Furthermore, detailed instructions were provided alongside the questionnaire to aid respondents in comprehending the research background and questions. Following the survey, a data cleaning process was implemented to eliminate samples with subpar answer quality, including those with short completion times or inconsistent responses. Subsequently, 475 high-quality valid questionnaires were acquired, yielding an effective response rate of approximately 86.4%. This sample size proves adequate to support the subsequent data analysis. The survey adhered strictly to research ethics, maintaining respondent anonymity to safeguard personal information and responses, and securing their informed consent for participation. Table 5 displays the demographic information of the respondents.

Table 5． Demographic information of respondents

Sample	Category	Frequency	Percentage
Age	55-60	138	29.1%
	61-65	179	37.7%
	66-70	95	20.0%
	＞70	63	13.3%

Gender	Male	233	49.1%
	Female	242	50.9%
Living status	not living with children	156	32.8%
	Living with children	319	67.2%
Education	Junior high school and below	224	47.2%
	High school and above	251	52.8%
Work status	Full-time work	102	21.5%
	Part-time work	115	24.2%
	Retired	231	48.6%
	never worked	27	5.7%
Primary means of living	Salary/wages	127	26.7%
	Property income	151	31.8%
	Alimony and/or government subsidies	197	41.5%
Occupation	Private-owned enterprise	190	40.0%
	Foreign-capital enterprise	116	24.4%
	Public sector or state-owned enterprises	47	9.9%
	liberal professions	122	25.7%

5. Data Analysis

5.1 Reliability and Validity Test

The data's reliability and validity underwent scrutiny via SPSS 24.0 and Smart PLS 4.0 software. The findings presented in Table 6 reveal that the Cronbach's alpha coefficients surpass 0.7 for all constructs, affirming the questionnaire's high reliability and facilitating subsequent analysis [63]. Furthermore, the Average Variance Extracted (AVE) exceeds 0.5 for each construct, denoting favorable convergent validity within the measurement model [64].

Table 6. Standardized factor loadings, Cronbach's alphas, CRs and AVEs

Construct	Item	Factor loading	Cronbach's alpha	rho_A	Composite reliability	AVE
Perceived Usefulness	PU 1	0.848	0.912	0.914	0.935	0.741
	PU 2	0.854				
	PU 3	0.854				
	PU 4	0.880				
	PU 5	0.867				
Perceived Ease of Use	PEOU 1	0.846	0.901	0.901	0.926	0.716
	PEOU 2	0.832				
	PEOU 3	0.864				
	PEOU 4	0.848				
	PEOU 5	0.840				
Social	SI 1	0.874	0.895	0.896	0.927	0.761

Influence	SI 2	0.865				
	SI 3	0.879				
	SI 4	0.872				
Facilitating Conditions	FC 1	0.871	0.888	0.891	0.922	0.748
	FC 2	0.856				
	FC 3	0.863				
	FC 4	0.870				
Perceived Trust	PT 1	0.879	0.860	0.860	0.914	0.781
	PT 2	0.892				
	PT 3	0.880				
Perceived Cost	PC 1	0.875	0.896	0.897	0.928	0.762
	PC 2	0.870				
	PC 3	0.888				
	PC 4	0.860				
Compatibility	COM 1	0.855	0.909	0.911	0.932	0.734
	COM 2	0.860				
	COM 3	0.851				
	COM 4	0.874				
	COM 5	0.843				
Behavioral Intention	BI 1	0.843	0.908	0.909	0.931	0.731
	BI 2	0.847				
	BI 3	0.860				
	BI 4	0.867				
	BI 5	0.858				

Based on previous investigations, it is imperative that all values within the Heterotrait-Monotrait (HTMT) ratio be below 0.90 [26, 65]. As delineated in Table 7, the findings affirm the adherence of these constructs to the standards of discriminant validity. Divergent validity was scrutinized to ascertain the absence of inter-measure correlation, with Table 8 presenting the resultant outcomes. Notably, the diagonal elements, indicative of the square root of Average Variance Extracted (AVE), demonstrate heightened correlation levels between any two specific factors. Consequently, a substantial majority of constructs within this study demonstrate commendable discriminant validity [66].

Table 7. Heterotrait-monotrait tatio (HTMT)

**Construct**	BI	COM	FC	PC	PEOU	PT	PU	SI
Behavioral Intention								
Compatibility	0.534							
Facilitating Conditions	0.525	0.468						
Perceived Cost	0.546	0.509	0.444					
Perceived Ease of Use	0.511	0.561	0.526	0.519				
Perceived Trust	0.446	0.521	0.444	0.463	0.531			

Perceived Usefulness	0.513	0.453	0.430	0.516	0.471	0.550		
Social Influence	0.504	0.517	0.524	0.503	0.540	0.525	0.489	

Table 8. Correlation matrix among constructs and square root of AVEs

**Construct**	BI	COM	FC	PC	PEOU	PT	PU	SI
Behavioral Intention	**0.855**							
Compatibility	0.487	**0.857**						
Facilitating Conditions	0.473	0.421	**0.865**					
Perceived Cost	-0.495	-0.460	-0.396	**0.873**				
Perceived Ease of Use	0.463	0.510	0.471	-0.466	**0.846**			
Perceived Trust	0.396	0.462	0.389	-0.406	0.468	**0.884**		
Perceived Usefulness	0.469	0.414	0.389	-0.469	0.428	0.488	**0.861**	
Social Influence	0.455	0.467	0.467	-0.452	0.486	0.461	0.444	**0.872**

5.2 Model Testing

This study utilized Smart PLS 4.0 to test hypotheses and validate the model. Previous research suggests an R^2 value of at least 0.26 as reasonable for explaining variance in the dependent variable, while in exploratory studies, an R^2 above 0.20 is considered acceptable [67]. The findings of this study indicate that the R^2 values for Behavioral Intention, Perceived Usefulness, and Perceived Ease of Use exceed 0.20, signifying their adequate explanation within the model. Scholars stress that a Q^2 value above zero validates the model's predictive relevance [68, 69]. The Q^2 values from this study meet the criteria, confirming the predictive relevance of the model. Detailed model fit indices are presented in Table 9.

Table 9. Model Fit Indices

**Construct**	**R^2**	**Q^2**
Behavioral Intention	0.415	0.298
Perceived Ease of Use	0.340	0.239
Perceived Usefulness	0.340	0.247

Additionally, an alternative approach to assess the goodness of fit (GoF) has been proposed by earlier scholars [26, 70]. The expression for calculating the GoF value is delineated below:

$$GoF = \sqrt{\overline{\text{AVE}} \times \overline{R^2}} = \sqrt{0.747 \times 0.365} \approx 0.522$$

The obtained GoF value in this investigation is 0.522, surpassing the recommended

threshold of 0.36 as proposed by Tenenhaus et al. [70] and Wei et al. [26], thereby affirming the model's validity.

The hypothesis was tested using Smart PLS 4, employing the bootstrapping technique [71]. Consistent with prior research standards, an SRMR < 0.08 is deemed acceptable; our study yielded an SRMR value of 0.058 [26]. The Variance Inflation Factor (VIF) remains a prevalent measure for assessing multicollinearity levels among variables [68]. As depicted in Table 10, the VIF values in this study fall within the range of 1.216 to 1.697, all below the threshold of 5 [67]. This confirms the absence of collinearity issues in the estimation model, thereby enhancing the reliability of its estimates.

The outcomes of path effects within this model are delineated in Table 10 and Figure 2. Except for the hypothesis H9 (Perceived Trust's effect on Behavioral Intention), all other pathways in the model demonstrated statistical significance. This underscores the meaningful contribution of this model towards elucidating the determinants influencing elderly people's intentions to adopt smart home systems.

Table 10. Model Path Analysis Results

Hypothesis	Path	Standardized coefficient (β)	t-Statistics	*p-Value*	VIF	Hypothesis status
H1	PEOU→PU	0.119	2.330	0.020	1.673	✔
H2	PU→BI	0.170	3.947	0.000	1.562	✔
H3	PEOU→BI	0.103	2.055	0.040	1.697	✔
H4	FC→PU	0.108	2.238	0.025	1.476	✔
H5	FC→PEOU	0.312	8.044	0.000	1.216	✔
H6	FC→BI	0.195	4.686	0.000	1.447	✔
H7	SI→PU	0.162	3.468	0.001	1.600	✔
H8	PT→PU	0.266	5.594	0.000	1.500	✔
H9	PT→BI	0.026	0.568	0.570	1.577	✘
H10	PC→BI	-0.198	4.332	0.000	1.552	✔
H11	COM→PU	0.110	2.261	0.024	1.585	✔
H12	COM→PEOU	0.378	9.709	0.000	1.216	✔
H13	COM→BI	0.179	3.931	0.000	1.617	✔

Figure 2. Model Path Analysis Results

Note: *p < 0.05, ***p < 0.001. The red dashed line represents the invalid hypothesis.

6. Discussion

6.1 Direct Influencing Factors on Elderly People's Behavioral Intention

6.1.1 Direct Positive Influencing Factors

The study findings suggest that facilitating conditions exert the most substantial positive influence on elderly people's behavioral intention toward smart home systems (supporting H6), with a path coefficient of 0.195. This reaffirms the central role of

facilitating conditions as proposed in the Unified Theory of Acceptance and Use of Technology. This outcome aligns with the investigations conducted by Chen and Chan [39] and Li et al. [24]. Furthermore, compatibility and perceived usefulness exert positive influences on behavioral intention (supporting H13 and H2). Li et al. [24] and Pal et al. [35] have both validated the significant influence of compatibility on behavioral intention. Additionally, Pal et al. [35] illustrated the positive impact of perceived usefulness on behavioral intention within the domain of smart home services. Moreover, perceived ease of use also exerts a positive influence on behavioral intention (supporting H3). This reaffirms the pivotal role of perceived ease of use as posited in the Technology Acceptance Model. In addition, findings from Song et al. [37] and Wei et al. [26] support the significant positive influence of perceived ease of use on behavioral intention.

6.1.2 Direct negative Influencing Factor

Perceived cost exerts a significant negative influence on behavioral intention, with a path coefficient of -0.198 (supporting H10). The rising expenses associated with implementing smart home systems might adversely impact the mindset of the elderly populace, potentially inducing resistance due to excessive financial burdens. Research by Pal et al. [43] substantiates that perceived cost indeed negatively affects behavioral intention toward smart homes for elderly healthcare.

6.2 Indirect Influencing Factors on Elderly People's Behavioral Intention

6.2.1 Factors Influencing Perceived Ease of Use

Social influence, perceived trust, facilitating conditions, compatibility, and perceived ease of use all indirectly influence behavioral intention through perceived usefulness. Notably, perceived trust has the most significant impact on behavioral intention through perceived usefulness, with coefficients of 0.266 (supporting H8). These findings are consistent with previous research. Pal et al. [43] suggested that an increase in perceived trust enhances the performance expectancy of elderly users' acceptance of smart homes. Given that perceived usefulness in the Technology Acceptance Model corresponds to performance expectancy in the Unified Theory of Acceptance and Use of Technology [42], it can be inferred that perceived trust positively influences perceived usefulness.

Social influence exerts a significant positive impact on behavioral intention through perceived usefulness, with coefficients of 0.162 (supporting H7). This finding is in line with prior studies such as Li et al. [24], which confirmed the positive influence of social influence on perceived usefulness within the smart wearable acceptance model. Additionally, perceived ease of use indirectly influences behavioral intention through perceived usefulness (supporting H1). This reaffirms the assumed relationships among these factors within the technology acceptance model framework.

Furthermore, compatibility also indirectly affects behavioral intention through perceived usefulness (supporting H11). This result aligns with previous research in the field. In the context of smart home systems, evaluating technical interoperability with

contemporary devices like smartphones and wireless sensor networks is crucial for determining the capacity to transmit monitoring data to remote devices. Prior studies by Pal et al. [35] and Li et al. [24] have further confirmed the positive impact of compatibility on perceived usefulness.

Moreover, facilitating conditions indirectly influence behavioral intention through perceived usefulness (supporting H4). This finding is consistent with the research conducted by Chen and Chan [39], which demonstrated that facilitating conditions positively influence perceived usefulness.

6.2.2 Factors Influencing Usefulness

Facilitating conditions exert a positive impact on behavioral intention through perceived ease of use, with coefficients of 0.312 (supporting H5). This aligns with previous research findings. Chen and Chan [39] conducted a study confirming the substantial impact of facilitating conditions on PEOU within the senior technology acceptance model. Likewise, Li et al. [24] demonstrated a favorable relationship between facilitating conditions and both perceived ease of use within the context of the smart wearable acceptance model. Furthermore, compatibility positively influences behavioral intention through perceived ease of use, with coefficients of 0.378 (supporting H12). This finding further substantiates previous research in the field. The studies conducted by Li et al. [24] have confirmed the significant influence of compatibility on perceived ease of use.

6.3 The Invalid Hypothesis

The results indicate that the direct effect of perceived trust on behavioral intention is not supported, with coefficients of 0.026 (not supporting H9). This is inconsistent with prior research findings. Research by Pal et al. [43] has demonstrated that perceived trust positively impacts behavioral intention in the field of smart home healthcare for the elderly. The inconsistency stems from differences in the study population; smart home elderly healthcare and the subjects of this study may possess different characteristics and needs. Additionally, although perceived trust does not directly influence behavioral intention, it exerts an indirect effect on behavioral intention through perceived usefulness. This implies that in this study, elderly people's perceived trust in the smart system does not directly translate into support for behavioral intention, but rather indirectly influences it through perceived usefulness.

7. Conclusion

The results demonstrate that facilitating conditions are the most significant positive direct influencing factor, playing a central role in smart home systems for the elderly. Therefore, in designing and promoting smart home systems for the elderly, emphasis should be placed on providing appropriate guidance, services, and training to strengthen facilitating conditions. Meanwhile, perceived cost has been confirmed as an important negative direct influencing factor, indicating the significance of pricing in the elderly's selection of smart home systems. Additionally, compatibility, perceived usefulness, and

perceived ease of use also exert positive direct effects on behavioral intention. Particularly noteworthy is that compatibility and facilitating conditions generate significant indirect positive effects on behavioral intention through perceived ease of use, while perceived trust influences behavioral intention indirectly through perceived usefulness. Therefore, future smart home systems for the elderly should enhance device connectivity and provide convenient usage conditions to improve perceived ease of use among the elderly. Simultaneously, attention should be given to issues of trust among the elderly regarding smart home systems, which would help enhance perceived usefulness and consequently promote behavioral intention. Furthermore, perceived usefulness is influenced by social influence, perceived ease of use, compatibility, and facilitating conditions, underscoring the crucial mediating role of perceived usefulness in the model. Thus, future system designs and developments should consider these factors. Although the direct impact of perceived trust on behavioral intention has not been confirmed, it may be because in the field of smart home systems for the elderly, perceived trust tends to exert indirect rather than direct effects on behavioral intention. Future research could further explore this aspect.

Through in-depth discussion and analysis of behavioral intentions towards Smart home systems for elderly people, this study has drawn several pivotal conclusions, providing practical strategies to enhance elderly people's willingness to adopt smart home systems:

Focus on facilitating conditions: The research findings indicate that facilitating conditions are the most significant positive direct influencing factor. Therefore, future designs and promotions of smart home systems should prioritize facilitating conditions. Firstly, more intuitive and understandable user guides should be provided. As elderly people may not be familiar with technical operations, clear and concise user guides are crucial for them. These guides should explain the system's functions and operation steps in simple language, helping them to quickly familiarize themselves and make full use of the system. Secondly, providing comprehensive services and training is also crucial. System providers should consider conducting regular training activities to help elderly people understand the system's functions and address any issues or confusion they encounter during usage.

Optimize pricing strategies: The study demonstrates that perceived cost is one of the key factors for the elderly to consider when adopting smart home systems. Therefore, future smart home system manufacturers should thoroughly understand the price expectations of elderly people and develop pricing strategies accordingly based on market positioning. Elderly people typically tend to be more cautious about the costs of purchasing any product or service. Since their income is relatively fixed after retirement, they pay more attention to the impact of product prices on their financial situation. Therefore, the price of smart home systems should be within their budget range to encourage consideration for purchase. Additionally, if the elderly perceive that the price is disproportionate to the functionality, they may refrain from purchasing. Manufacturers should ensure that smart home systems provide sufficient value and

convenient features to balance the relationship between price and performance.

Enhance device connectivity and usability: Compatibility and facilitating conditions indirectly influence behavioral intention through perceived ease of use. Therefore, in future designs of smart home systems for the elderly, it is important to enhance connectivity between different devices, ensuring that users can easily integrate and control various smart devices. Furthermore, providing clear and comprehensive operation manuals and technical training is crucial. This can help elderly users become more familiar with and proficient in using the system, enhancing their perceived ease of use and, consequently, their intention to use the system.

Address trust issues: Trust issues among elderly people significantly affect perceived usefulness of smart home systems. Therefore, future research and designs of smart home systems for the elderly should focus on issues of perceived trust to promote perceived usefulness among elderly users and subsequently encourage adoption. Specifically, trust can be strengthened by providing simple and user-friendly interfaces, regular technical training, ensuring privacy security, and implementing effective feedback mechanisms to enhance understanding and interaction with smart home systems, gradually building trust.

In conclusion, this study provides valuable insights into the determinants of elderly people's intention to adopt smart home systems. It underscores the importance of factors such as facilitating conditions, perceived cost, perceived trust, compatibility, and perceived usefulness, shedding light on their pivotal role in the decision-making dynamics of elderly people.

7.1 Theoretical Contributions

This study makes significant theoretical contributions to the field of smart homes for the elderly. Firstly, by integrating the Technology Acceptance Model and the Unified Theory of Acceptance and Use of Technology, this study constructed a theoretical model of user behavioral intention for smart home systems among the elderly, providing a profound analysis of the decision-making mechanisms involved in the elderly's acceptance of technology. The research validated the predominant role of facilitating conditions, perceived cost, perceived trust, compatibility, and perceived usefulness as core influencing factors on the behavioral intention of the elderly, emphasizing the need for comprehensive consideration of these factors in the development and design of smart home systems for the elderly. This theoretical model provides robust support for a deeper understanding of the acceptance mechanisms of artificial intelligence technology in the field of smart home systems for the elderly.

7.2 Practical Implications

On a practical level, this study provides crucial practical guidance for stakeholders to promote the application and acceptance of smart home systems among the elderly. Addressing elderly people specifically, the research emphasizes the importance of

providing smart home systems that are easier to understand and use, suggesting the reduction of product prices and the enhancement of training and services to increase their adoption willingness and confidence. For smart home system developers and designers, the research findings suggest a focus on facilitating conditions, perceived cost, compatibility, and user experience. This directs investment towards improving connectivity and usability between devices, formulating practical market positioning and pricing strategies to better attract and meet the needs of the elderly. As for government and relevant agencies, the study calls for proactive promotion of smart home system development through policy support, standardized regulation, and provision of educational resources, ensuring information security and privacy protection for the elderly, promoting societal digital inclusivity, enabling elderly people to better integrate into the digital society, and enjoy the convenience and benefits brought by technology.

7.3 Research Limitations and Future Research

While this study provides in-depth insights into the behavioral intention of the elderly towards smart home systems, there are still some limitations. Firstly, the sample primarily consisted of people from economically developed regions along the eastern coast of China. This geographical and demographic limitation constrains the generalizability of the research findings. Future studies should consider expanding the sample scope to cover a broader range of areas and backgrounds to comprehensively understand the behavioral intention of the elderly towards smart home systems across different regions.

Moreover, the study employed a cross-sectional design, which failed to capture the long-term evolution and dynamic changes in the elderly's behavioral intention towards smart home systems. Future research could consider longitudinal study designs to track changes in the elderly's behavioral intention towards smart home systems, providing a more comprehensive understanding of its developmental trends and shifts in user attitudes.

Furthermore, this study utilized quantitative research methods to validate the model of smart home systems for the elderly. However, future research could extend to qualitative or mixed research methods. Qualitative research can delve deeper into the real needs and expectations of the elderly towards smart home systems, gathering specific insights through interviews, focus groups, etc., to design systems that are more aligned with the lifestyles and habits of the elderly. Mixed research methods, combining the strengths of qualitative and quantitative research, can verify the model while gaining a deeper understanding of the motivations and meanings behind the elderly's behavior, thus obtaining a more comprehensive understanding.

Lastly, this study primarily focused on the factors influencing the elderly's intention to use smart home systems without specific research on different types of smart home systems. Future research could explore how different types of smart home systems

affect the behavioral intention of the elderly, providing more specific guidance and recommendations for developers and designers.

Declarations

Author contributions： Conceptualization: Y. W., N. M. S. Data Curation: Y. W. and Y.H. H. Formal Analysis and Investigation: Y. W. and Y.H. H. Methodology: Y. W. Software: Y. W. Supervision: N. M. S., Y.H. H. Writing – Original Draft Preparation: Y. W. Writing – Review & Editing: N. M. S., Y.H. H., Q.L. J. and L. Z.

Declaration of Interest statement: The authors have declared that no competing interests exist.

Funding Disclosure: This research was conducted without any external funding.

Ethics Declarations: This study was reviewed and approved by Qufu Normal University Biomedical Ethics Committee, with the approval number: 2024051. All respondents provided written informed consent to participate in the study.

Data Availability Statement： To protect the privacy of the respondents, the data supporting the findings of this study are available upon request from the corresponding author, the data supporting the findings of this study are available upon request from the corresponding author.

References

1. Alvarez, P. *Charted: The World's Aging Population from 1950 to 2100*. Visual Capitalist 2023; Available from: https://www.visualcapitalist.com/cp/charted-the-worlds-aging-population-1950-to-2100/.
2. Sina. *The largest 'retirement wave' is approaching, and someone has done the math*. 2022; Available from: https://finance.sina.com.cn/money/fund/jjzl/2022-08-11/doc-imizmscv5829962.shtml.
3. NBS. *Bulletin of the Seventh National Census (No. 5)*. 2021; Available from: https://www.stats.gov.cn/sj/tjgb/rkpcgb/qgrkpcgb/202302/t20230206_1902005.html.
4. Song, J. and D. Ryu, *Aging effects on consumption risk-sharing channels in European countries.* Zbornik radova Ekonomskog fakulteta u Rijeci: časopis za ekonomsku teoriju i praksu, 2018. **36**(2): p. 585-617.
5. Marois, G., A. Bélanger, and W. Lutz, *Population aging, migration, and productivity in Europe.* Proceedings of the National Academy of Sciences, 2020. **117**(14): p. 7690-7695.DOI: 10.1073/pnas.1918988117.
6. Cristea, M., et al., *The Impact of Population Aging and Public Health Support on EU Labor Markets.* International Journal of Environmental Research and Public Health, 2020. **17**(4): p. 1439.DOI: 10.3390/ijerph17041439.
7. SOHU. *The 2023 "age standard for older adults" is announced, and the World Health Organization redefines young people*. 2023; Available from: https://www.sohu.com/a/717847668_121687419.
8. Shankar, A., et al., *Loneliness, social isolation, and behavioral and biological health indicators in older adults.* Health psychology, 2011. **30**(4): p. 377.DOI: 10.1037/a0022826.
9. Steptoe, A., et al., *Social isolation, loneliness, and all-cause mortality in older men and women.* Proceedings of the National Academy of Sciences, 2013. **110**(15): p. 5797-5801.DOI: 10.1073/pnas.1219686110.
10. Machielse, J., *Afgezonderd of ingesloten? Over sociale kwetsbaarheid van ouderen.* 2016.
11. Guner, H. and C. Acarturk, *The use and acceptance of ICT by senior citizens: a comparison of technology acceptance model (TAM) for elderly and young adults.* Universal Access in the Information Society, 2020. **19**(2): p. 311-330.DOI: 10.1007/s10209-018-0642-4.
12. Carnemolla, P., *Ageing in place and the internet of things – how smart home technologies, the built environment and caregiving intersect.* Visualization in Engineering, 2018. **6**(1): p. 7.DOI: 10.1186/s40327-018-0066-5.
13. Yuan, Y., et al., *Baseline Survey of China Social Work Longitudinal Study 2019: Design and Implementation.* Research on Social Work Practice, 2021. **31**(5): p. 513-519.DOI: 10.1177/1049731520984536.
14. Choukou, M.-A. and S. Syed-Abdul, *Smart Home Technologies and Services for Geriatric Rehabilitation*. 2021: Academic Press. 256.
15. Dermody, G., et al., *Factors influencing community-dwelling older adults' readiness to adopt smart home technology: A qualitative exploratory study.* Journal of Advanced Nursing, 2021. **77**(12): p. 4847-4861.DOI: 10.1111/jan.14996.
16. Hong, Y.K., Z.Y. Wang, and J.Y. Cho, *Global Research Trends on Smart Homes for Older Adults: Bibliometric and Scientometric Analyses.* International Journal of Environmental Research and Public Health, 2022. **19**(22).DOI: 10.3390/ijerph192214821.

17. De Silva, L.C., C. Morikawa, and I.M. Petra, *State of the art of smart homes.* Engineering Applications of Artificial Intelligence, 2012. **25**(7): p. 1313-1321.DOI: 10.1016/j.engappai.2012.05.002.
18. Junestrand, S. and K. Tollmar. *Video mediated Communication for domestic environments: Architectural and technological design*. 1999. Springer.
19. Alaa, M., et al., *A review of smart home applications based on Internet of Things.* Journal of network and computer applications, 2017. **97**: p. 48-65.DOI: 10.1016/j.jnca.2017.08.017.
20. Keeler, L.W. and M.J. Bernstein, *The future of aging in smart environments: Four scenarios of the United States in 2050.* Futures, 2021. **133**: p. 102830.DOI: 10.1016/j.futures.2021.102830.
21. Zeng, F. and T.-L. Chen, *A study of the acceptability of smart homes to the future elderly in China.* Universal Access in the Information Society, 2023. **22**(3): p. 1007-1025.DOI: 10.1007/s10209-022-00871-1.
22. Ji, W. and E.H.W. Chan, *Critical Factors Influencing the Adoption of Smart Home Energy Technology in China: A Guangdong Province Case Study.* Energies, 2019. **12**(21): p. 4180.DOI: 10.3390/en12214180.
23. Zhang, Q., M. Li, and Y. Wu, *Smart home for elderly care: development and challenges in China.* BMC Geriatrics, 2020. **20**(1): p. 318.DOI: 10.1186/s12877-020-01737-y.
24. Li, J., et al., *Health monitoring through wearable technologies for older adults: Smart wearables acceptance model.* Applied Ergonomics, 2019. **75**: p. 162-169.DOI: 10.1016/j.apergo.2018.10.006.
25. Arthanat, S., H. Chang, and J. Wilcox, *Determinants of information communication and smart home automation technology adoption for aging-in-place.* Journal of Enabling Technologies, 2020. **14**(2): p. 73-86.DOI: 10.1108/JET-11-2019-0050.
26. Wei, W.J., et al., *A study on community older people's willingness to use smart home-an extended technology acceptance model with intergenerational relationships.* FRONTIERS IN PUBLIC HEALTH, 2023. **11**.DOI: 10.3389/fpubh.2023.1139667.
27. Chiu, C.-J., S. Hsieh, and C.-W. Li, *Needs and Preferences of Middle-Aged and Older Adults in Taiwan for Companion Robots and Pets: Survey Study.* Journal of Medical Internet Research, 2021. **23**(6): p. e23471.DOI: 10.2196/23471.
28. He, J.P. and X. Huang, *The smartphone use and eudaimonic well-being of urban elderly: based on intergenerational support and TAM.* Chin. J. Commun, 2020. **3**: p. 49-73.
29. Zhou, C., et al., *Impact of swiping direction on the interaction performance of elderly-oriented smart home interface: EEG and eye-tracking evidence.* FRONTIERS IN PSYCHOLOGY, 2023. **14**.DOI: 10.3389/fpsyg.2023.1089769.
30. Zhong, R.T., et al., *User acceptance of smart home voice assistant: a comparison among younger, middle-aged, and older adults.* UNIVERSAL ACCESS IN THE INFORMATION SOCIETY, 2022.DOI: 10.1007/s10209-022-00936-1.
31. Forestal, R.L. and E.Y. Li. *Gerontechnology acceptance of smart homes: A systematic review and meta-analysis*. in *Proceedings of the International Conference on Electronic Business (ICEB)*. 2022.
32. Li, X. *Research on Design Strategy of Computer User Interface Based On Smart Home for the Elderly*. in *Journal of Physics: Conference Series*. 2021.
33. Ma, Q., A.H.S. Chan, and K. Chen, *Personal and other factors affecting acceptance of smartphone technology by older Chinese adults.* Applied Ergonomics, 2016. **54**: p. 62-71.DOI:

10.1016/j.apergo.2015.11.015.
34. Pal, D., et al., *Smart Homes and Quality of Life for the Elderly: Perspective of Competing Models.* IEEE Access, 2018. **6**: p. 8109-8122.DOI: 10.1109/ACCESS.2018.2798614.
35. Pal, D., et al., *Analyzing the Elderly Users' Adoption of Smart-Home Services.* IEEE Access, 2018. **6**: p. 51238-51252.DOI: 10.1109/ACCESS.2018.2869599.
36. Etemad-Sajadi, R. and G. Gomes Dos Santos, *Senior citizens' acceptance of connected health technologies in their homes.* International Journal of Health Care Quality Assurance, 2019. **32**(8): p. 1162-1174.DOI: 10.1108/IJHCQA-10-2018-0240.
37. Song, Y., Y. Yang, and P. Cheng, *The Investigation of Adoption of Voice-User Interface (VUI) in Smart Home Systems among Chinese Older Adults.* Sensors, 2022. **22**(4).DOI: 10.3390/s22041614.
38. Yan, J.J.K. and A.S.H. Lee. *A Conceptual Model on Integration of Technology Acceptance Model on Smart Home Healthcare System among Elderly*. in *CTISC 2022 - 2022 4th International Conference on Advances in Computer Technology, Information Science and Communications*. 2022.
39. Chen, K. and A.H. Chan, *Gerontechnology acceptance by elderly Hong Kong Chinese: a senior technology acceptance model (STAM).* Ergonomics, 2014. **57**(5): p. 635-52.DOI: 10.1080/00140139.2014.895855.
40. Arar, M., et al., *Analysis of Smart Home Technology Acceptance and Preference for Elderly in Dubai, UAE.* Designs, 2021. **5**(4): p. 70.DOI: 10.3390/designs5040070.
41. Tipaldi, P. and M. Natter, *Older adults' decisions on smart home systems: Better put an age tag on it!* Psychology and Marketing, 2022. **39**(9): p. 1747-1761.DOI: 10.1002/mar.21698.
42. Maswadi, K., N.A. Ghani, and S. Hamid, *Factors influencing the elderly's behavioural intention to use smart home technologies in Saudi Arabia.* PLoS ONE, 2022. **17**(8 August).DOI: 10.1371/journal.pone.0272525.
43. Pal, D., et al., *Internet-of-Things and Smart Homes for Elderly Healthcare: An End User Perspective.* IEEE Access, 2018. **6**: p. 10483-10496.DOI: 10.1109/ACCESS.2018.2808472.
44. Pal, D., et al., *Embracing the Smart-Home Revolution in Asia by the Elderly: An End-User Negative Perception Modeling.* IEEE Access, 2019. **7**: p. 38535-38549.DOI: 10.1109/ACCESS.2019.2906346.
45. Wadu Mesthrige, J., O.T. Oladinrin, and L.D. Ojo, *Critical Barriers of Using Smart Home Technologies (SHTs) to the Elderly in Hong Kong.* Journal of Aging and Environment, 2023. **37**(2): p. 219-244.DOI: 10.1080/26892618.2022.2062805.
46. Davis, F.D., *Perceived usefulness, perceived ease of use, and user acceptance of information technology.* MIS quarterly, 1989: p. 319-340.
47. Venkatesh, V., et al., *User acceptance of information technology: Toward a unified view.* MIS quarterly, 2003: p. 425-478.DOI: 10.2307/30036540.
48. Kim, S., et al., *Analysis of the factors influencing healthcare professionals' adoption of mobile electronic medical record (EMR) using the unified theory of acceptance and use of technology (UTAUT) in a tertiary hospital.* BMC Medical Informatics and Decision Making, 2016. **16**(1): p. 12.DOI: 10.1186/s12911-016-0249-8.
49. Deng, Z., X. Mo, and S. Liu, *Comparison of the middle-aged and older users' adoption of mobile health services in China.* Int J Med Inform, 2014. **83**(3): p. 210-24.DOI: 10.1016/j.ijmedinf.2013.12.002.

50. Jen, W.-Y. and M.-C. Hung, *An empirical study of adopting mobile healthcare service: the family's perspective on the healthcare needs of their elderly members.* Telemedicine and e-Health, 2010. **16**(1): p. 41-48.DOI: 10.1089/tmj.2009.0093.
51. Leung, K.F., et al., *Development and validation of the interview version of the Hong Kong Chinese WHOQOL-BREF.* Quality of Life Research, 2005. **14**(5): p. 1413-1419.DOI: 10.1007/s11136-004-4772-1.
52. Brown, S.A. and V. Venkatesh, *A Model of Adoption of Technology in the Household: A Baseline Model Test and Extension Incorporating Household Life Cycle.* Management Information Systems Quarterly, 2005. **29**(3): p. 11.DOI: 10.2307/25148690.
53. Ryu, M.-H., S. Kim, and E. Lee, *Understanding the factors affecting online elderly user's participation in video UCC services.* Computers in Human Behavior, 2009. **25**(3): p. 619-632.DOI: 10.1016/j.chb.2008.08.013.
54. Yang, H., et al., *User acceptance of wearable devices: An extended perspective of perceived value.* Telematics and Informatics, 2016. **33**(2): p. 256-269.DOI: 10.1016/j.tele.2015.08.007.
55. Yusif, S., J. Soar, and A. Hafeez-Baig, *Older people, assistive technologies, and the barriers to adoption: A systematic review.* International Journal of Medical Informatics, 2016. **94**: p. 112-116.DOI: 10.1016/j.ijmedinf.2016.07.004.
56. Islam, M.M. and M.E. Hossain, *An Investigation of Consumers' Acceptance of Mobile Banking in Bangladesh.* International Journal of Innovation in the Digital Economy (IJIDE), 2015. **6**(3): p. 16-32.DOI: 10.4018/ijide.2015070102.
57. Ram, S. and J.N. Sheth, *Consumer Resistance to Innovations: The Marketing Problem and its solutions.* Journal of Consumer Marketing, 1989. **6**(2): p. 5-14.DOI: 10.1108/EUM0000000002542.
58. Chiu, C.-Y., C.-L. Chen, and S. Chen, *Broadband Mobile Applications' Adoption by SMEs in Taiwan—A Multi-Perspective Study of Determinants.* Applied Sciences, 2022. **12**(14): p. 7002.
59. Birch, A. and V. Irvine, *Preservice teachers' acceptance of ICT integration in the classroom: Applying the UTAUT model.* Educational media international, 2009. **46**(4): p. 295-315.
60. Wrycza, S., B. Marcinkowski, and D. Gajda, *The enriched UTAUT model for the acceptance of software engineering tools in academic education.* Information systems management, 2017. **34**(1): p. 38-49.
61. Cohen, J., *Statistical power analysis for the behavioral sciences (revised ed.)*. 1977: New York: Academic Press.
62. Sivo, S.A., et al., *How low should you go? Low response rates and the validity of inference in IS questionnaire research.* Journal of the association for information systems, 2006. **7**(1): p. 17.
63. Hair, J.F., et al., *Multivariate data analysis*. 2006: NJ: Pearson Prentice Hall.
64. Hair Jr, J.F., et al., *Partial Least Squares Structural Equation Modeling (PLS-SEM) Using R: A Workbook*. 2021: Springer Nature.
65. Henseler, J., C.M. Ringle, and M. Sarstedt, *A new criterion for assessing discriminant validity in variance-based structural equation modeling.* Journal of the Academy of Marketing Science, 2015. **43**(1): p. 115-135.DOI: 10.1007/s11747-014-0403-8.
66. Fornell, C. and D.F. Larcker, *Evaluating structural equation models with unobservable variables and measurement error.* Journal of marketing research, 1981. **18**(1): p. 39-50.
67. Hair Jr, J., et al., *A primer on partial least squares structural equation modeling (PLS-SEM)*. 2021: Sage publications.

68. Henseler, J., C.M. Ringle, and R.R. Sinkovics, *The use of partial least squares path modeling in international marketing*, in *New challenges to international marketing*. 2009, Emerald Group Publishing Limited. p. 277-319.
69. Hair, J.F., et al., *A Primer on Partial Least Squares Structural Equation Modeling (PLS-SEM).* 2017.
70. Tenenhaus, M., et al., *PLS path modeling.* Computational Statistics & Data Analysis, 2005. **48**(1): p. 159-205.DOI: 10.1016/j.csda.2004.03.005.
71. Bollen, K.A. and R.A. Stine, *Bootstrapping Goodness-of-Fit Measures in Structural Equation Models.* Sociological Methods & Research, 1992. **21**(2): p. 205-229.DOI: 10.1177/0049124192021002004.

Figure 1

Click here to access/download;Figure;Figure 1.tif

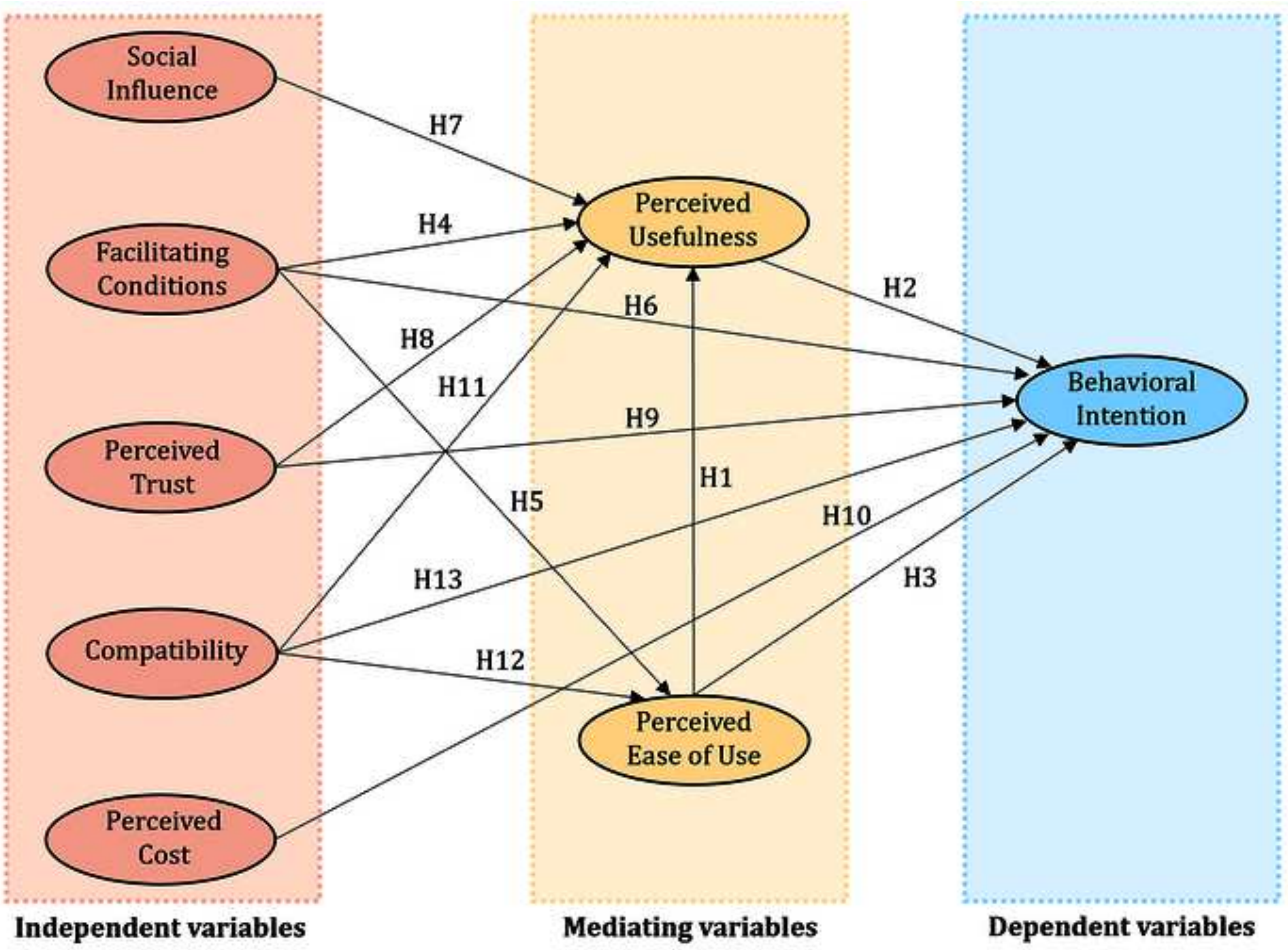

Figure 2

Click here to access/download;Figure;Figure 2.tif

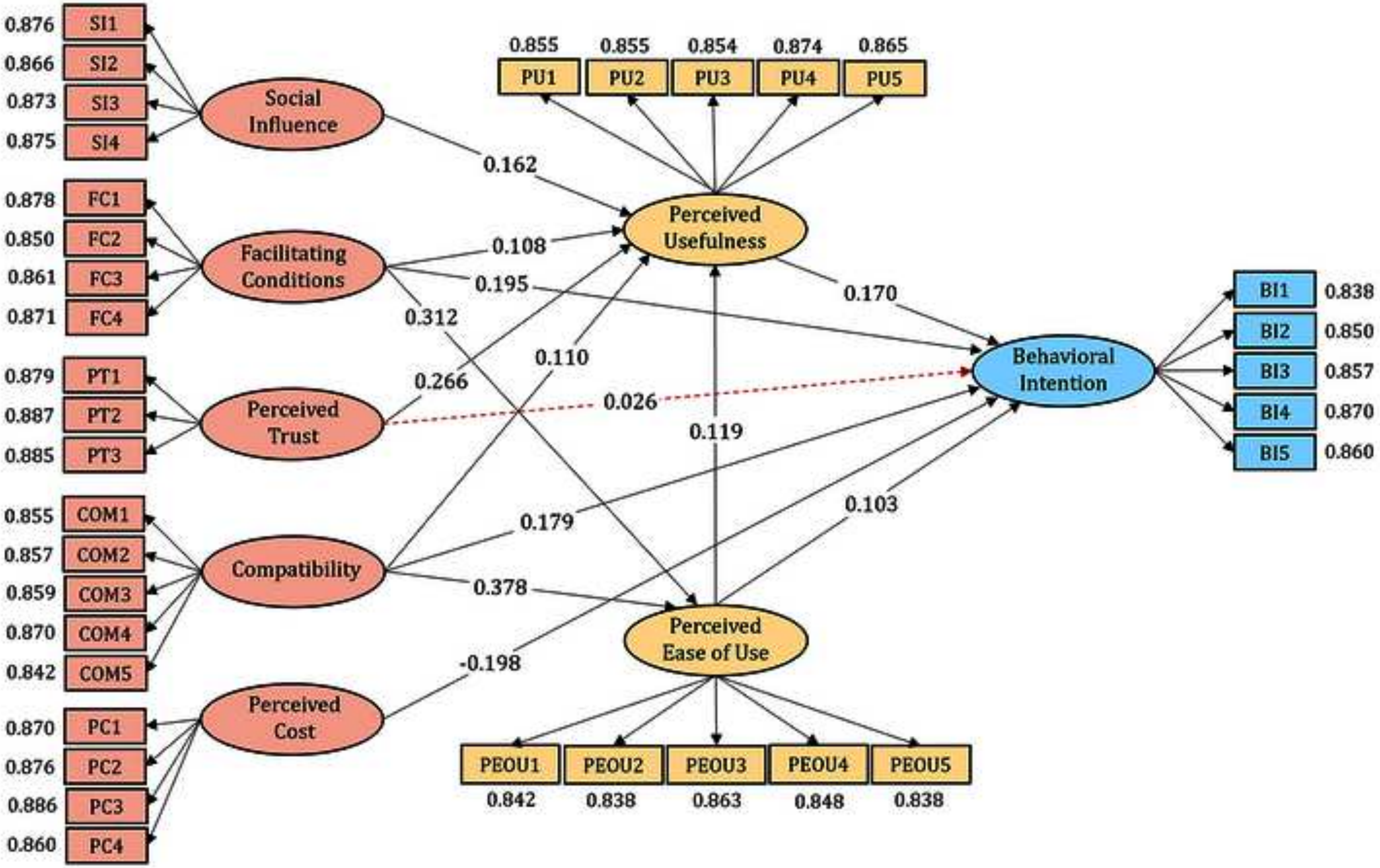

Supplement: Attachment — Submitted filename: PONE-D-24-20607.pdf [file pone.0311280.s002.pdf]
