# Supplementary material for: Factors affecting the elderly’s behavioral intention toward smart home systems: A cross-sectional study from China’s eastern coast
Source: PLoS One. 2024 Dec 13;19(12):e0311280. doi: 10.1371/journal.pone.0311280 (PMC11643254; doi:10.1371/journal.pone.0311280)
Supplement: Attachment — Submitted filename: Reviewer Report_PONE-D-24-20607.docx [file pone.0311280.s003.docx]

**Reviewer Report _** **PONE-D-24-20607**

Thanks for giving me a chance to review this manuscript. This is an interesting topic. The author(s) tries to work significantly. *This study aims to address this gap by constructing a theoretical model of elderly user behavioral intention, drawing upon the Technology Acceptance Model (TAM) and the Unified Theory of Acceptance and Use of Technology (UTAUT)*. However, still, some of the anomalies I found during the review process are addressed below, which may help further develop the study.

1. **Originality**

It can be improved

-	The author(s) should illustrate how this research differs from the literature in the field / or considered a contribution to the literature.

-	For the **practical study gap**, the author (s) should focus on the concrete details of the situation in the **Chinese** context:

(a.	Where and when does the problem arise? b. Who does the problem affect? c. What attempts have been made to solve the problem?)

-	For **theoretical research**, the author (s) should re-think about the scientific, social, geographical, and/or historical background:

(a.	What is already known about the problem? b. Is the problem limited to a certain time period or geographical area? c. How has the problem been defined and debated in the scholarly literature?)

The abovementioned issues could help to improve the originality of this study and make it stronger for interested readers, researchers, and/or practitioners in the field.

- The author(s) need to illustrate how their work could contribute to the knowledge in the field and how their work differs from previously published studies

2. **Relationship to Literature**

It can be improved

- In the “literature review” section, the author(s) should review the main sub-themes in this study topic. This section is recommended to conclude how the reviewed literature is different from the current study and how this study could contribute to the research area.
- The “literature review” should represent a survey of scholarly sources on this study topic. It should provide an overview of current knowledge, allowing both readers and authors to identify relevant theories, methods, and gaps in the existing research that could later be applied to this research work. **I believe that the authors should improve their “literature review” section to reflect that.**
- **The hypotheses development paragraphs should be improved; (further discussion should be added)!**
- **A sub-section “theoretical background and hypotheses development should be added to section 2. (please restructure the section)**
- **A more up-to-date studies should be cited and discussed (especially those which based on UTAUT and TAM), for example:**

Alkhwaldi, Abeer F., and Asem S. Al Eshoush. "Towards a model for citizens’ acceptance of e-payment systems for public sector services in Jordan: evidence from crisis era." Information Sciences Letters 11, no. 3 (2022): 657-663.

Alwadain, A., Fati, S. M., Ali, K., & Ali, R. F. (2024). From theory to practice: An integrated TTF-UTAUT study on electric vehicle adoption behavior. Plos one, 19(3), e0297890.

Alkhwaldi, A. F., & Abdulmuhsin, A. A. (2022). Understanding user acceptance of IoT based healthcare in Jordan: Integration of the TTF and TAM. In Digital economy, business analytics, and big data analytics applications (pp. 191-213). Cham: Springer International Publishing.

Zhou, C., Qian, Y., & Kaner, J. (2024). A study on smart home use intention of elderly consumers based on technology acceptance models. Plos one, 19(3), e0300574.

3. **Methodology**:

Yes, the method is appropriate. **However**, it could be improved as follows:

-	Usually, the research methodology section recommended to include:

1.	The type of research you conducted

2.	How you collected and analyzed your data

3.	Any tools or materials you used in the research

4.	How do you mitigate or avoid research biases

5.	Why did you choose these methods

Please make sure that the “methodology” section includes the above-mentioned elements

- Justify the use of the questionnaire
- Why **China** was selected?
- Justify the selection of this region “the eastern coastal regions of China.
- “stratified sampling approach” justifies the use of this approach
- **Justify the use of SEM and PLS**

4. **Results**

- The results discussion should be linked to the previous studies in the field

**General Comments:**

- At the end of the “introduction section”, the author(s) should include how this manuscript has been organized! (the content of each section)
- Both “theoretical and practical implications” should be improved and linked to the findings of the current study.
- The author(s) should be clear about which implications reflect the practical perspective and which reflect the theoretical perspective

**Quality of Communication:**

-	There is a need for proofreading and professional editing.
