# Supplementary material for: Factors affecting the elderly’s behavioral intention toward smart home systems: A cross-sectional study from China’s eastern coast
Source: PLoS One. 2024 Dec 13;19(12):e0311280. doi: 10.1371/journal.pone.0311280 (PMC11643254; doi:10.1371/journal.pone.0311280)
Supplement: Attachment — Submitted filename: Response to Reviewers.docx [file pone.0311280.s004.docx]

Dear Editor and Reviewers of PLOS ONE,

On behalf of my co-authors, I would like to express our sincere gratitude for the thoughtful comments and suggestions provided on our manuscript, titled “Factors Affecting Elderly Behavioral Intention Towards Smart Home Systems: A Cross-Sectional Study from China’s Eastern Coast” (Manuscript Number: PONE-D-24-20607). We have carefully considered the feedback and made revisions accordingly, which we hope will meet your expectations.

To facilitate your review, we have made the revisions visible using the “Track Changes” mode in the “Revised Manuscript with Track Changes.” The changes are annotated as follows：

- Fonts: Text that has been removed

- Fonts: Newly inserted text

- Fonts: Text that has been relocated

Additionally, our responses to the Editor’s and Reviewers’ comments are highlighted in the “Revised Manuscript with Track Changes.” The specific highlights are as follows:

- Fonts: Response to Editor’s comments

- Fonts: Response to Reviewer 1’s comments

- Fonts: Response to Reviewer 2’s comments

Below is our point-by-point response to each comment from the Editor and Reviewers. The issues raised by the Editor and Reviewers are highlighted in black font, while our responses are presented in dark blue italics.

# Response to the Editor’s comments:

*Thank you for taking the time to review our manuscript and for your valuable suggestions. In the “Revised Manuscript with Track Changes,” we have highlighted our responses to your comments in gray for your convenience.*

1. The introduction must be enhanced by a clearer statement of the research gap and the study's specific objectives.

***Our response:***

*Thanks very much for your nice comments. We agree that the introduction must be enhanced by a clear statement of the research gap and objectives. The modified content is as follows:*

*“Despite the significant improvements that smart home systems can offer to the elderly [25-28], their adoption rate in this demographic remains relatively low [36, 37]. Therefore, it is essential to explore the key factors influencing the behavioral intentions of older adults. Although existing studies have extensively examined the technical aspects of smart home systems [21, 38, 39], there is a significant lack of empirical research from a user perspective, particularly in the Chinese context. This study aims to investigate the behavioral intentions of the elderly in the eastern coastal regions of China toward smart home systems” (Page 7, Line 134-144).*

2. In the introduction section, it could be beneficial to provide more specific statistics or references related to the elderly population in China's eastern coastal regions.

***Our response:***

*Thank you for your valuable suggestion. In response, we have expanded the introduction to include a more detailed description of the aging population in China’s eastern coastal regions, based on the most recent census data. Furthermore, we have created Figure 1, which presents the data for the ten provinces with the largest aging populations in China. Most of these provinces are concentrated in the eastern coastal regions.* *The modified content is as follows：*

*“According to the ‘Bulletin of the Seventh National Population Census,’ China has the world’s largest elderly population, particularly concentrated in the eastern coastal provinces, such as Shandong, Jiangsu, Guangdong, Hebei, and Zhejiang [4]. Fig 1 A presents the top 10 provinces by elderly population, and Fig 1 B illustrates their geographical locations” (Page 4, Line 79- Page 5, Line 83).*

*Fig 1. The top 10 provinces in China with the largest elderly populations in 2020.*

*(A)	Number of elderly people in the top 10 provinces, (B) The geographical locations of these provinces (Page 5, Line 97- 100).*

3. (1) The integration of TAM and UTAUT in your research model needs stronger justification. Discuss the theoretical and practical considerations for using specific constructs from each model. Include any pilot study results that support your choice.

***Our response:***

*Thank you for your insightful comments, which we fully agree with. Our literature review confirmed that TAM and UTAUT are the most widely adopted theoretical models in this field of research. Specifically, the variables of TAM (perceived usefulness and perceived ease of use) and UTAUT (social influence and facilitating conditions) are frequently applied in studies examining the acceptance of smart home technology among the elderly. Moreover, we selected TAM and UTAUT because perceived usefulness and perceived ease of use have moderating effects between the UTAUT variables, extended variables, and behavioral intention. The modified content is as follows：*

*“In the context of smart home systems for the elderly, Pal et al. [55] examined the factors influencing the adoption of smart home services by the elderly using the TAM. Etemad-Sajadi and Gomes Dos Santos [56] explored the use of IoT health technologies in the homes of older adults guided by the TAM. Yan & Lee [57] developed a model for a smart home healthcare system for the elderly, grounded in based on the TAM. These studies demonstrate that perceived usefulness and perceived ease of use have strong explanatory power for the elderly’s adoption of smart home technologies and devices. Although multiple studies indicate that the TAM is applicable in the context of smart homes for the elderly, scholars have pointed out that it overlooks the influence of social factors on the technology adoption process [65]. This limitation could be overcome by incorporating external variables” (Page 14, Line 265- Page 15, Line 285)*

*“Notably, the UTAUT includes social influence, thus addressing the limitations of the TAM, which tends to overlook social factors” (Page 16, Line 304- 306).*

*“The UTAUT has also been widely used in studies related to smart homes for older adults. Scholars have pointed out that the UTAUT model was initially developed based on systems in workplace environments, in which the determining factors may differ from those in a home setting [65]. However, many empirical studies on UTAUT have been conducted in home environments [44, 45, 50, 54, 55, 59-61]. Arar et al. [59] used the UTAUT framework to analyze the acceptance and preferences of older adults in Dubai, UAE, toward smart home technology. Maswadi et al. [61] employed the UTAUT model to study the behavioral intentions of the elderly in Saudi Arabia to adopt smart home technologies. Zhong et al. [50] used the UTAUT framework to investigate differences in the acceptance of smart home voice assistants across age groups. These studies largely identified social influence and facilitating conditions as key variables and validated their effectiveness” (Page 16, Line 310- Page 17, Line 326).*

*“Several scholars have integrated variables from the TAM and UTAUT for empirical research [44, 53, 58]. Researchers have combined variables such as social influence, facilitating conditions, perceived usefulness, perceived ease of use, and compatibility to construct a comprehensive model examining older adults’ behavioral intentions toward smart wearable devices [44]. The results indicated that perceived usefulness and perceived ease of use mediated the relationship between various factors and behavioral intention, thus enhancing the understanding of user behavioral intentions” (Page 18, Line 363- Page 19, Line 369).*

(2) The substitution of performance expectancy and effort expectancy with perceived usefulness and perceived ease of use is not clearly explained. These constructs are conceptually similar, so explain why the TAM constructs offer unique advantages.

***Our response:***

*Thank you for highlighting this point. We have addressed it by adding a subsection titled “A Comparison between the TAM and UTAUT” within the “Literature Review” section to provide a detailed explanation. The details are as follows:*

*“A comparison between the TAM and UTAUT shows that the perceived usefulness in the TAM corresponds to performance expectancy in the UTAUT, whereas perceived ease of use corresponds to effort expectancy [61, 81]. Perceived usefulness and perceived ease of use were selected as the research variables for two main reasons. First, compared with performance expectancy and effort expectancy, perceived usefulness and perceived ease of use have been widely applied in studies on the acceptance of smart home technologies, confirming their applicability in the smart home domain [10, 23, 46, 57]. Second, multiple studies have confirmed the mediating effects of perceived usefulness and perceived ease of use on other research variables and behavioral intention [82-84]. The mediating roles of perceived usefulness and perceived ease of use allow for a more comprehensive understanding of the complex mechanisms underlying the behavioral intentions of the elderly” (Page 17, Line 329- Page 18, Line 345).*

4. The hypotheses section is currently shallow and lacks clarity. It would benefit from a more structured and detailed presentation. Each hypothesis should be separately stated and explicitly linked to the theoretical constructs, supported by recent references from the last 3 years.

***Our response:***

*Thank you for your detailed suggestions regarding the hypothesis section.* *We have thoroughly revised it to improve clarity. To enhance the structure, we have introduced subheadings in the “Hypotheses Development” section, specifically “Variables of the TAM,” “Variables of the UTAUT,” and “Extended Variables.” Under these subheadings, we have elaborated on the research variables and linked them to their corresponding theoretical constructs. Additionally, we have separated the previously grouped hypotheses, presenting each one individually. Given the length of this section, we have not included it here. However, in the “Revised Manuscript with Track Changes,” these modifications are highlighted in gray for your review (Page 22, Line 426- Page 32, Line 641).*

*We have also updated this section with the latest literature. In the “Revised Manuscript with Track Changes,” the new and removed references are marked in different colors in the “Reference” section for clarity. New references, numbered 68-77, 79-80, 82-84, 90-92, 94-97, and 99-103, 106-109, are highlighted in blue. Additionally, we have removed some older references, numbered 85-87, 89, 98, 104, and 105, which are marked in red (Page 82, Line 1637- Page 85, Line 1787).*

5. Subsection 3.3 Model Construction is not needed. Consider providing a visual model of the theoretical framework (Figure 1) early in the section to guide readers before the hypotheses are developed.

***Our response:***

*Thanks very much for your nice comments. We have removed “Subsection 3.3 Model Construction” (Page 32, Line 643- Page 33, Line 666)* *and integrated a visual model of the theoretical framework into the earlier “Theoretical background”* *section (Page 20, Line 392-394). Consequently, the numbering of the figures has been updated, with the new image now labeled as “Fig 2,” as detailed below.*

***Fig 2. Theoretical framework.***

6. (1) In the Methodology section, the description of the sample size calculation can be streamlined for better clarity and conciseness. (2) Additionally, please provide more detailed information on the pilot study, including the number of participants, the feedback received, and how the suggestions from domain experts were incorporated into the final questionnaire design.

***Our response:***

*Thank you for your comments. We fully agree with your suggestions and have revised the “Sample Size” section to enhance clarity and brevity. Due to the length of this section, the details are not listed here. Please refer to the “Sample Size” section in the “Revised Manuscript with Track Changes” for a comprehensive view of the modifications (Page 39, Line 737- Page 40, Line 762).*

(2) Additionally, please provide more detailed information on the pilot study, including the number of participants, the feedback received, and how the suggestions from domain experts were incorporated into the final questionnaire design.

***Our response:***

*We appreciate your suggestion and fully agree with it. We’ve also included a “Pilot Study” section that details the number of participants, the feedback received, and how the experts’ suggestions were integrated into the final questionnaire design. The modified content is as follows:*

*“A pilot study and expert validation were conducted before collecting data [61]. In this pilot study, 35 participants from diverse backgrounds were invited to evaluate the reliability and representativeness of the questionnaire. After completing the questionnaire, the respondents provided feedback on content relevance, language clarity, and comprehension difficulty. In addition, three experts in smart home technology were consulted to review the questionnaires. They provided in-depth suggestions regarding the structure and content, including the accuracy of wording, professionalism of the language, and optimization of the question order. Based on the feedback from both experts and participants, comprehensive adjustments were made to the questionnaire to ensure its suitability for subsequent data collection” (Page 38, Line 712- 722).*

7. Add a new subsection within the Methodology section to justify using PLS-SEM in your study. This subsection should elaborate on why PLS-SEM is the most appropriate analytical technique for your research.

***Our response:***

*Thank you for your valuable feedback, which has been instrumental in improving our manuscript. In response, we have incorporated a new subsection titled “PLS-SEM” within the “Research Methodology” section, in which we have provided a detailed explanation of our rationale for selecting PLS-SEM. The revised content is presented below:*

*“This study used PLS-SEM for data analysis based on three key considerations. First, compared with CB-SEM, PLS-SEM is more suitable for non-normally distributed data. Because data in this study did not follow a normal distribution, the selection of PLS-SEM ensured the robustness of the analysis and predictive capability of the model [111]. Second, PLS-SEM emphasizes the predictive power and explanatory ability of the model, making it particularly well-suited for models involving multiple variables and complex relationships [112]. As the model in this study involved complex relationships between several variables, PLS-SEM was considered more appropriate. Additionally, PLS-SEM has been widely applied in this field, providing a reference and guidance for our methodological choice [46, 57, 61]” (Page 34, Line 680- 689).*

8. In survey research, positional bias can occur, leading to skewed results. It is crucial to discuss the procedures used to mitigate this bias within your study. Please add a new subsection in the analysis section to address this issue.

***Our response:***

*Thank you for your insightful advice. In response, we have added a subsection titled “Mitigation of Positional Bias” in the “Data Analysis and Results” section to address this issue. The revised content is provided below:*

*“Position bias refers to a phenomenon in which user behavior and selection outcomes are influenced by the positions of items or options on a page or list [122]. To address this issue, a small-scale pilot study was conducted by randomizing the order of the questions and options in the questionnaire to assess potential position bias. The order of the questions and scale items was adjusted based on participant feedback and expert recommendations, and neutral wording was used to minimize language bias. These steps were taken to reduce the impact of position bias on the study results” (Page 46, Line 865- Page 47, 872).*

9. The discussion could be more focused. Focus on the most critical results and their implications. Differentiate between direct and indirect effects and their practical significance.

***Our response:***

*Thank you for your valuable suggestion, which we have taken into careful consideration. In response, we have thoroughly reorganized and revised the “Discussion” section, addressing key hypotheses within the newly structured “Findings of the Direct Hypotheses” and “Findings of the Mediating Hypotheses” sections. Due to the length of the content, we are unable to list it here, but you can find the detailed revisions in the “Discussion” section of the “Revised Manuscript with Track Changes” (Page 54, Line 976- Page 62, Line 1131).*

*Additionally, we have revised the descriptions of the direct and indirect effects, as well as their practical implications, in the “Conclusion” section as follows：*

*“This study clarified the distinct mechanisms by which direct and indirect factors influenced behavioral intentions among the elderly. Direct influencing factors have a straightforward relationship with behavioral intention, making them suitable for scenarios in which a short-term effect is required in the early stages of product promotion. By contrast, indirect influencing factors affect behavioral intention through mediating variables, making them more applicable to long-term promotion and user development strategies. The practical implications of these findings are explored from the perspectives of the elderly, developers, designers, and policymakers…” (Page 70, Line 1304- Page 73, Line 1356).*

10. The theoretical contributions and practical implications sections require further development. In the theoretical contributions, there is a need for a deeper exploration of how your findings specifically contribute to these theories. Regarding the practical implications, please provide actionable recommendations for smart home system developers, designers, policymakers, and other stakeholders.

***Our response:***

*We appreciate your suggestion and have made extensive revisions accordingly. Based on our findings, we have rewritten the “Theoretical contributions” and “Practical implications” sections. In terms of theoretical contributions, we validated the adaptability of TAM and UTAUT in the context of elderly acceptance of smart home systems, with the inclusion of external variables expanding the existing theoretical model. Additionally, we confirmed the mediating role of perceived usefulness and perceived ease of use between UTAUT variables, external variables, and behavioral intention. The specific details are as follows:*

*“This study examines the key factors influencing older adults’ intentions to use smart home systems, deepens our understanding of their behavioral intentions, and expands the theoretical framework of technology acceptance among the elderly.*

*First, by integrating the TAM, UTAUT, and their extended variables, this study developed a comprehensive model that revealed the complex mechanisms driving older adults’ behavioral intentions toward smart home systems. The findings demonstrate that perceived usefulness, facilitating conditions, compatibility, and perceived cost have significant direct effects on behavioral intention. This validates the applicability of the TAM and UTAUT in this context and underscores the importance of the extended variables (compatibility and perceived cost) in influencing behavioral intention, thereby enriching the existing theoretical framework.*

*In addition, this study explored the mediating effects of perceived ease of use and perceived usefulness. The results demonstrated that facilitating conditions and compatibility indirectly influenced behavioral intention through perceived ease of use, whereas social influence and perceived trust indirectly affected behavioral intention through perceived usefulness. These findings revealed the complex mechanisms underlying the formation of behavioral intentions among the elderly, offering new perspectives for understanding their intentions toward smart home systems and expanding the theoretical model” (Page 68, Line 1264- Page 69, Line 1284).*

*In the “Practical implications” section, we have distinguished between hypotheses with direct and indirect effects on behavioral intention and summarized their practical implications from the perspectives of various stakeholders (the elderly, developers, designers, and policymakers). Due to the length, the detailed modifications are not included here. For a full overview, please refer to the “Practical implications” section in the “Revised Manuscript with Track Changes” (Page 69, Line 1286- Page 73, Line 1356).*

11. There are many grammatical errors throughout the manuscript. A thorough proofreading is recommended.

***Our response:***

*Thank you very much for your suggestion, which we greatly appreciate.* *We carefully checked the manuscript and polished it in full through the professional polishing agency “editage.” The polishing certificate is included in the “Appendix” section at the end of the document, please check it out.*

12. Ensure all references are complete and formatted according to the Plos One’s guidelines.

***Our response:***

*We appreciate your valuable feedback and the time you dedicated to reviewing our manuscript. In response to your suggestion, we carefully reviewed PLOS ONE’s formatting guidelines and made comprehensive revisions to the “References” section. Specifically, we updated the reference style from “Numbered” to the “Vancouver” format and adjusted the DOI entries from the “URL” format to the standard DOI format. We have also ensured the accurate display of the first six authors and standardized the formats for different types of references. The specific modifications can be found in the “References” section (Page 77, Line 1425- Page 87, Line 1860).*

# Response to the Reviewer 1’s comments:

*Thank you for your thorough review and valuable suggestions on our manuscript. In the “Revised Manuscript with Track Changes,” we have highlighted our responses to your comments in yellow. However, where our responses overlap with previous replies to the Editor (originally highlighted in gray), we have maintained the gray highlights for consistency. We apologize for any inconvenience this may cause. To assist with your review, we have also included specific page and line numbers in the text following each response.*

1. Introduction:

The introduction needs to be harmonised, there are too many paragraphs.

***Our response:***

*Thank you for your valuable suggestion. We have carefully reorganized and rewritten the “Introduction” section, condensing the original four paragraphs into three. In response to feedback from other reviewers, we also added a paragraph outlining the article’s structure. Due to the length of this section, we have not provided the details here. For specific modifications, please refer to the “Introduction” section in the “Revised Manuscript with Track Changes” (Page 4, Line 68- Page 8, Line 155).*

2. Related Work:

The objectives should be in the introduction section, not in the literature review.

***Our response:***

*Thank you for your kind comments. We have relocated the research objective to the “Introduction” section. As the editor also pointed out this issue, this part has been highlighted in gray. The revised content is as follows:*

*“This study aims to investigate the behavioral intentions of the elderly in the eastern coastal regions of China toward smart home systems” (Page 7, Line 142- 144).*

3. Theoretical Framework and Research Hypotheses

The authors need to explain the strength and weakness of the chosen theory, and justify why chosen it for the study.

***Our response:***

*We appreciate your insightful feedback and agree with your suggestions. In response, we have thoroughly revised the theoretical background section to include a detailed discussion of the advantages and disadvantages of TAM and UTAUT, along with the rationale for selecting these models. The revised content is as follows:*

***Advantages of TAM:***

*“The TAM is a highly reliable and empirically supported theoretical model [65]. Derived from TRA, the TAM focuses on understanding users’ behavioral intentions to voluntarily use information technology systems [66]” (Page 13, Line 243-245).*

*“Today, the TAM is recognized as one of the most popular and persuasive models for studying user acceptance of information technology [10, 65]” (Page 13, Line 257-259).*

*“The TAM has been widely applied across various fields including education [68, 69], business [70-72], computer science and information systems [73-75], and green sustainable technology [76, 77]. In the context of smart home systems for the elderly, Pal et al. [55] examined the factors influencing the adoption of smart home services by the elderly using the TAM…* *These studies demonstrate that perceived usefulness and perceived ease of use have strong explanatory power for the elderly’s adoption of smart home technologies and devices” (Page 14, Line 261- Page 15, Line 282).*

***Disadvantages of TAM:***

*“Although multiple studies indicate that the TAM is applicable in the context of smart homes for the elderly, scholars have pointed out that it overlooks the influence of social factors on the technology adoption process [65]” (Page15, Line 282-285).*

***Advantages of UTAUT:***

*“The UTAUT is derived from the integration of eight theories: the Theory of Planned Behavior (TPB), TAM, combination of TAM and TPB, TRA, Model of Personal Computer Utilization, Diffusion of Innovation Theory, Social Cognitive Theory and Motivation Model [78]” (Page 15, Line 298- Page 16, Line 302).*

*“Notably, the UTAUT includes social influence, thus addressing the limitations of the TAM, which tends to overlook social factors” (Page 16, Line 304- 306).*

*“The UTAUT has been applied in various fields, such as healthcare [79], smart technology [59, 61], mobile commerce [70], higher education [79], and electric vehicles [80]. The UTAUT has also been widely used in studies related to smart homes for older adults” (Page 16, Line 308- 311).*

***Disadvantages of UTAUT:***

*“Scholars have pointed out that the UTAUT model was initially developed based on systems in workplace environments, in which the determining factors may differ from those in a home setting [65]” (Page 16, Line 311- 313).*

***Rationale for Selecting TAM and UTAUT：***

*As previously mentioned, TAM and UTAUT each have distinct advantages and are widely applied in the study of smart homes for the elderly. The variables from TAM (perceived usefulness, perceived ease of use, and behavioral intention) and UTAUT (social influence, facilitating conditions, and behavioral intention) have been shown to significantly impact the acceptance of smart homes among the elderly. Furthermore, UTAUT's “social influence” variable addresses the absence of social factors in TAM. Additionally, research indicates that perceived usefulness and perceived ease of use mediate the relationship between various factors and behavioral intention.*

*In this field of research, numerous empirical studies have integrated TAM and UTAUT, as demonstrated below：*

*“Several scholars have integrated variables from the TAM and UTAUT for empirical research [44, 53, 58]. Researchers have combined variables such as social influence, facilitating conditions, perceived usefulness, perceived ease of use, and compatibility to construct a comprehensive model examining older adults’ behavioral intentions toward smart wearable devices [44]. The results indicated that* *perceived usefulness and perceived ease of use mediated the relationship between various factors and behavioral intention, thus enhancing the understanding of user behavioral intentions” (Page 18, Line 363- Page 19, Line 369).*

4. Methodology:

The methodology needs corrections regard which research designed employed and all the section needs to be re structure.

***Our response:***

*Thank you for your thoughtful comments. We have reorganized this section after carefully reviewing relevant papers.*

*First, we have added a description of the research methods, as follows：*

*“This study used structural equation modeling (SEM) for quantitative research. As a multivariate analysis tool, SEM is widely used across various fields because of its ability to comprehensively examine complex relationships between variables [110]. Researchers in this field have adopted SEM for their analyses [10, 46, 50, 61]. As this study aimed to validate the relationships between multiple independent and dependent variables, this method was considered appropriate. SEM includes models based on partial least squares (PLS-SEM) and covariance-based (CB-SEM) [110]. PLS-SEM typically employs Smart PLS software for data analysis, whereas CB-SEM is conducted using Amos” (Page 33, Line 669- Page 34, Line 677).*

*Second, we have included additional sections on “PLS-SEM,” “Pilot Study,” “Inclusivity in Global Research,” and “Informed Consent” to provide detailed explanations of our research tools, pilot study procedures, and ethical considerations. Due to space constraints, we have not listed them here. For detailed information, please refer to the respective sections in the “Revised Manuscript with Track Changes.” The corresponding page and line numbers are provided below：*

*-PLS-SEM：Page 34, Line 679- 689*

*-Pilot study：Page 38, Line 712- 722*

*-Inclusivity in global research：Page 42, Line 788- 791*

*-Informed consent：Page 42, Line 800- Page 43, Line 816*

*Moreover, we revised the term “Questionnaire Design” to “Survey instrument” for greater clarity (Page 34, Line 691).*

5. Results:

The result is well presented.

***Our response:***

*Thank you for your positive feedback and support.*

6. Conclusion:

The conclusion section is well-structured and effectively summarizes the key findings of the study.

***Our response:***

*Thank you for your valuable feedback and the time and effort you dedicated to reviewing this manuscript.*

7. General Comments

In general, the authors need to proofread the whole article, and check the grammar.

***Our response:***

*We agree with you and believe it is essential. After thoroughly revising and reviewing the manuscript, we had it professionally edited by “editage.” The polishing certificate is included in the “Appendix” section at the end of the document, please check it out.*

# Response to the Reviewer 2’s comments:

Thanks for giving me a chance to review this manuscript. This is an interesting topic. The author(s) tries to work significantly. This study aims to address this gap by constructing a theoretical model of elderly user behavioral intention, drawing upon the Technology Acceptance Model (TAM) and the Unified Theory of Acceptance and Use of Technology (UTAUT). However, still, some of the anomalies I found during the review process are addressed below, which may help further develop the study. (Comments are included in the attached file.)

***Our response:***

*Thank you for your thorough and professional review of our manuscript. In response to your valuable suggestions, we have made extensive revisions. In the “Revised Manuscript with Track Changes,” we have highlighted our responses to your questions in blue. For sections where your comments overlapped with previous feedback from the Editor and Reviewer 1, we have retained the original gray and yellow highlights for consistency. We apologize for any inconvenience this may cause. To assist with your review, we have also included specific page and line numbers in the text following each response.*

1. Originality

It can be improved.

(1) The author(s) should illustrate how this research differs from the literature in the field / or considered a contribution to the literature.

***Our response:***

*Thank you for your careful review and insightful suggestions. We agree with you and have accordingly made the necessary revisions. The differences and contributions of this study are now detailed in the following paragraph:*

*“Although existing studies have extensively examined the technical aspects of smart home systems [21, 38, 39], there is a significant lack of empirical research from a user perspective, particularly in the Chinese context.* *This study aims to investigate the behavioral intentions of the elderly in the eastern coastal regions of China toward smart home systems. The findings of this research not only provide theoretical insights into the behavioral intentions of the elderly but also offer practical guidance for developers, designers, and policymakers in their efforts to innovate, design, and promote smart home systems” (Page 7, Line 140- Page 8, Line 147).*

(2) For the practical study gap, the author (s) should focus on the concrete details of the situation in the Chinese context:

***Our response:***

*Thank you for your specific and comprehensive advice. After carefully considering your suggestions, we have reorganized and revised this section as follows:*

a. Where and when does the problem arise?

*“Since becoming an aging society in 2000, China’s aging process has rapidly accelerated, with the country expected to enter a stage of severe aging by 2035 [1]” (Page 4, Line 68-69).*

*“According to the ‘Bulletin of the Seventh National Population Census,’ China has the world’s largest elderly population, particularly concentrated in the eastern coastal provinces, such as Shandong, Jiangsu, Guangdong, Hebei, and Zhejiang [4]” (Page 4, Line 79- Page 5, Line 81).*

b. Who does the problem affect?

*“This trend poses significant challenges across the social, economic, healthcare, and elderly care sectors. Socially and economically, the increasing proportion of the elderly has exacerbated labor market tensions, affecting productivity and economic growth [6]. In healthcare, the risk of chronic diseases among the elderly has increased sharply, leading to a substantial increase in healthcare demands [7]. Additionally, the high proportion of one-child families in the eastern region intensifies the burden of elderly care [8]. Age-friendly housing and community planning have become crucial [9]” (Page 5, Line 83-90).*

c. What attempts have been made to solve the problem?

*“These issues have garnered significant attention from governments and academia, leading to the introduction of multiple policies [10]. Over the past decade, China has increased its investment in social welfare, encouraging the participation of various social forces to gradually establish a multi-party system for providing elderly care. With advancements in the Internet of Things (IoT), information technology, big data, and cloud computing, the Chinese government has been actively promoting smart elderly care [11]” (Page 5, Line 90-96).*

(3) For theoretical research, the author(s) should re-think about the scientific, social, geographical, and/or historical background:

***Our response:***

*We appreciate your feedback and fully agree with your points. Below are our detailed responses to each of your questions:*

a. What is already known about the problem?

*We have added the subsections “Smart home systems for the elderly” and “Theoretical background”* *to the “Literature review” section to address the current research status and problem. The details are as follows:*

*“In recent years, researchers have extensively explored the benefits of smart home systems for elderly populations. These studies primarily focused on advancements in health monitoring, voice control, and behavior prediction from the perspectives of technology and design methods…These studies have primarily focused on technical aspects, with limited research from a user perspective, particularly in the context of China” (Page 8, Line 160- Page 9, Line 175).*

*“Researchers have applied various theoretical models to studies related to smart homes for the elderly, such as the Technology Acceptance Model (TAM) [10, 46, 56, 57], Unified Theory of Acceptance and Use of Technology (UTAUT) [50, 59, 61], Theory of Planned Behavior [54], and Theory of Reasoned Action (TRA) [54]. These models aim to explain the factors driving individuals to adopt technology, with the TAM and UTAUT being the most widely used. However, most studies have focused on the use of smart home technologies and devices by the elderly, and there is a lack of research on smart home systems based on these theories” (Page 12, Line 232- Page 13, 239).*

b. Is the problem limited to a certain time period or geographical area?

*Research on smart home acceptance among older adults is not confined to specific regions. Our review revealed that scholars from various countries have explored this topic, for example, Arar et al. [59] used the UTAUT framework to analyze the acceptance and preferences of older adults in Dubai, UAE, toward smart home technology. Maswadi et al. [61] employed the UTAUT model to study the behavioral intentions of the elderly in Saudi Arabia to adopt smart home technologies.*

c. How has the problem been defined and debated in the scholarly literature?

*In the scholarly literature,* *it is widely recognized that smart home systems can enhance the independent living and overall well-being of elderly people. However, despite these potential benefits, the adoption rate of smart home systems among the elderly remains relatively low. The specifics are as follows:*

*“Leveraging the IoT, smart home systems integrate household devices, appliances, and security features into an automated and remotely controllable environment [20]. These technologies offer numerous benefits to the aging process, for example, safety monitoring, emergency alerts, and anti-intrusion features of smart home systems significantly enhance the sense of security among elderly residents [21]. In addition, smart climate control, lighting systems, and appliances can automatically adjust to the preferences and behavioral patterns of the elderly, thereby improving their overall comfort [22]. Moreover, smart home systems empower older adults to manage daily activities independently, such as controlling devices via voice commands and remotely monitoring their health, thereby reducing their reliance on others [23]” (Page 6, Line 111-121).*

*“Despite the significant improvements that smart home systems can offer to* *the elderly [25-28], their adoption rate* *in this demographic remains relatively low [36, 37]” (Page 7, Line 134-138).*

*Through the review, we found that most studies on the acceptance of smart technology by elderly users rely on the TAM and UTAUT models. However, there is ongoing debate among scholars regarding the applicability of these theories, as outlined below:*

*“The TAM has been widely applied across various fields including education [68, 69], business [70-72], computer science and information systems [73-75], and green sustainable technology [76, 77]. In the context of smart home systems for the elderly, Pal et al. [55] examined the factors influencing the adoption of smart home services by the elderly using the TAM…These studies demonstrate that perceived usefulness and perceived ease of use have strong explanatory power for the elderly’s adoption of smart home technologies and devices” (Page 14, Line 261- Page 15, 282).*

*“Although multiple studies indicate that the TAM is applicable in the context of smart homes for the elderly, scholars have pointed out that it overlooks the influence of social factors on the technology adoption process [65]” (Page 15, Line 282-285).*

*“The UTAUT has been applied in various fields, such as healthcare [79], smart technology [59, 61], mobile commerce [70], higher education [79], and electric vehicles [80]. The UTAUT has also been widely used in studies related to smart homes for older adults. Scholars have pointed out that the UTAUT model was initially developed based on systems in workplace environments, in which the determining factors may differ from those in a home setting [65]. However, many empirical studies on UTAUT have been conducted in home environments [44, 45, 50, 54, 55, 59-61]” (Page 16, Line 308-314).*

The abovementioned issues could help to improve the originality of this study and make it stronger for interested readers, researchers, and/or practitioners in the field.

*We appreciate and agree with your perspective.*

(4) The author(s) need to illustrate how their work could contribute to the knowledge in the field and how their work differs from previously published studies.

***Our response:***

*We appreciate and agree with your suggestion. We elaborate on the differences and contributions of this study from previous studies in the following paragraph:*

*“Although existing studies have extensively examined the technical aspects of smart home systems [21, 38, 39], there is a significant lack of empirical research from a user perspective, particularly in the Chinese context.* *This study aims to investigate the behavioral intentions of the elderly in the eastern coastal regions of China toward smart home systems. The findings of this research not only provide theoretical insights into the behavioral intentions of the elderly but also offer practical guidance for developers, designers, and policymakers in their efforts to innovate, design, and promote smart home systems” (Page 7, Line 140- Page 8, Line 147).*

2. Relationship to Literature

It can be improved

(1) a. In the “literature review” section, the author(s) should review the main sub-themes in this study topic.

***Our response:***

*We agree with and appreciate your suggestion. In the “Literature review” section, we have added subsections on “smart home systems for the elderly” and “Theoretical background” to thoroughly review the relevant topics of this study. The details are as follows：*

*“Smart home systems leverage the IoT technology to connect devices and appliances, thereby enabling automated management and remote control [20]. In recent years, researchers have extensively explored the benefits of smart home systems for elderly populations. These studies primarily focused on advancements in health monitoring, voice control, and behavior prediction from the perspectives of technology and design methods. From a technical standpoint, Zou et al. [39] designed a Raspberry Pi-based smart home system to detect fatigue levels in older adults and control appliances. Other scholars have examined the application of voice- control technology in smart home systems to enhance their ease of use by older users [40]. From a design perspective, Cui and Chung [41] analyzed the design methods for smart elderly care housing under the concept of “user-centered” design, emphasizing the importance of meeting user needs. Yun et al. [42] proposed an information fusion model for designing age-appropriate home environments and developed a behavioral prediction system for the elderly. Additionally, researchers have investigated the perceptions of smart home systems among the elderly in South Korea [43]. These studies have primarily focused on technical aspects, with limited research from a user perspective, particularly in the context of China” (Page 8, Line 158- Page 9, Line 175).*

*Furthermore, in the “Theoretical background” section, we have discussed TAM, UTAUT, and the extended variables. Given the length of this section, we have not included the details here. We kindly ask you to refer to the “Theoretical Background” section in the “Revised Manuscript with Track Changes” for further information (Page 12, Line 231- Page 20, Line 391).*

b. This section is recommended to conclude how the reviewed literature is different from the current study and how this study could contribute to the research area.

***Our response:***

*Through literature review, we found that current research on smart home systems for the elderly mainly focuses on the technical level and lacks research from the user perspective. The details are as follows:*

*“In recent years, researchers have extensively explored the benefits of smart home systems for elderly populations. These studies primarily focused on advancements in health monitoring, voice control, and behavior prediction from the perspectives of technology and design methods…These studies have primarily focused on technical aspects, with limited research from a user perspective, particularly in the context of China” (Page 8, Line 160- Page 9, 175).*

*In the “Theoretical background” section of the literature review, we also examine the relevant theoretical models in this field and highlight the differences between this study and existing research. The details are as follows:*

*“Researchers have applied various theoretical models to studies related to smart homes for the elderly, such as the Technology Acceptance Model (TAM) [10, 46, 56, 57], Unified Theory of Acceptance and Use of Technology (UTAUT) [50, 59, 61], Theory of Planned Behavior [54], and Theory of Reasoned Action (TRA) [54]. These models aim to explain the factors driving individuals to adopt technology, with the TAM and UTAUT being the most widely used…” (Page 12, Line 231- Page 13, Line 240).*

*“However, most studies have focused on the use of smart home technologies and devices by the elderly, and there is a lack of research on smart home systems based on these theories. To address this gap, this section constructs a comprehensive model based on the TAM, UTAUT, and extended variables” (Page 12, Line 237- Page 13, Line 240).*

*The contributions of this study are outlined in both the “Introduction” and “Conclusion” sections. In the “Literature Review” section, we focused specifically on the theoretical contribution, which involves the development of a comprehensive model that addresses the research gap in understanding the acceptance of smart home systems among older adults. The details are as follows:*

*“Despite the widespread application of the TAM and UTAUT in studies on smart home technologies and devices, research on the acceptance of smart home systems based on these theoretical models remains limited. Building on the TAM, UTAUT, and their extended variables, this study proposed a comprehensive theoretical framework (Fig 2)” (Page 20, Line 388- 391).*

(2) The “literature review” should represent a survey of scholarly sources on this study topic. It should provide an overview of current knowledge, allowing both readers and authors to identify relevant theories, methods, and gaps in the existing research that could later be applied to this research work. I believe that the authors should improve their “literature review” section to reflect that.

***Our response:***

*Thank you for your suggestion; we fully agree with it. We have reorganized and rewritten this section accordingly.*

*First, we added the subsections “Smart home systems for the elderly" and “theoretical background” to the “Literature review” section to provide a comprehensive overview of the current research status, relevant theories, methods, and existing gaps (Page 8, Line 157- Page 20, 391).*

*In addition, in our review of the “Smart home systems for the elderly” subsection, we found that most current research primarily focuses on the technical aspects, with limited attention given to the user perspective. The details are as follows:*

*“Smart home systems leverage the IoT technology to connect devices and appliances, thereby enabling automated management and remote control [20]. In recent years, researchers have extensively explored the benefits of smart home systems for elderly populations. These studies primarily focused on advancements in health monitoring, voice control, and behavior prediction from the perspectives of technology and design methods... These studies have primarily focused on technical aspects, with limited research from a user perspective, particularly in the context of China” (Page 8, Line 158- Page 9, Line 175).*

*Moreover, from a “Theoretical background” perspective, TAM and UTAUT are the most widely used models in this field. However, there are currently few empirical studies on the acceptance of smart home systems by the elderly within the Chinese context. Due to the length of the article, we have provided only a description of the research gap below:*

*“… Despite the widespread application of the TAM and UTAUT in studies on smart home technologies and devices, research on the acceptance of smart home systems based on these theoretical models remains limited” (Page 12, Line 231- Page 20, Line 391).*

(3) The hypotheses development paragraphs should be improved; (further discussion should be added)!

***Our response:***

*We appreciate your suggestion and have implemented the recommended changes. The section has been reorganized and rewritten as follows:*

*First, we added the heading “Hypotheses development”* *and aligned each variable with its respective theoretical model. Under the “Variables of the TAM” heading, we included “perceived ease of use,” “perceived usefulness,” and “behavioral intention.” The “Variables of the UTAUT” heading covers “facilitating conditions” and “social influence.” Additionally, we grouped “perceived trust,” “perceived cost,” and “compatibility” under the “Extended Variables” heading (Page 22, Line 426- Page 32, Line 641).*

*Furthermore, to enhance the clarity of the hypothesis discussion, we elaborated on each hypothesis individually and expanded the original discussion for each one (Page 22, Line 426- Page 32, Line 641).*

(4) A sub-section “theoretical background and hypotheses development should be added to section 2. (Please restructure the section).

***Our response:***

*We appreciate your suggestion and have incorporated it into our revisions. In response, we have added “Theoretical background” and “Hypotheses development” to the second part. Below are the corresponding page and line numbers in the “Revised Manuscript with Track Changes”:*

*- Theoretical background (Page 12, Line 231)*

*- Hypotheses development (Page 22, Line 426)*

(5) A more up-to-date studies should be cited and discussed (especially those which based on UTAUT and TAM), for example:

Alkhwaldi, Abeer F., and Asem S. Al Eshoush. "Towards a model for citizens’ acceptance of e-payment systems for public sector services in Jordan: evidence from crisis era." Information Sciences Letters 11, no. 3 (2022): 657-663.

Alwadain, A., Fati, S. M., Ali, K., & Ali, R. F. (2024). From theory to practice: An integrated TTF-UTAUT study on electric vehicle adoption behavior. Plos one, 19(3), e0297890.

Alkhwaldi, A. F., & Abdulmuhsin, A. A. (2022). Understanding user acceptance of IoT based healthcare in Jordan: Integration of the TTF and TAM. In Digital economy, business analytics, and big data analytics applications (pp. 191-213). Cham: Springer International Publishing.

Zhou, C., Qian, Y., & Kaner, J. (2024). A study on smart home use intention of elderly consumers based on technology acceptance models. Plos one, 19(3), e0300574.

***Our response:***

*We appreciate and agree with your suggestion. We have carefully reviewed, updated, and cited the mentioned documents, along with other relevant sources. The corresponding references are now numbered in the “Reference” section of the “Revised Manuscript with Track Changes” as follows:*

*70．* *Alkhwaldi AF, Eshoush. ASA. Towards A model for Citizens’ Acceptance of E-Payment Systems for Public Sector Services in Jordan: Evidence from Crisis Era. Information Sciences Letters. 2022; 11(3):657-63. doi: 10.18576/isl/110302. (In-text citation: Page 14, Line 264; Page 16, Line 309)*

*80．Alwadain A, Fati SM, Ali K, Ali RF. From theory to practice: An integrated TTF-UTAUT study on electric vehicle adoption behavior. PLOS ONE. 2024; 19(3): e0297890.doi: 10.1371/journal.pone.0297890. (In-text citation: Page 16, Line 310)*

*75．Alkhwaldi AF, Abdulmuhsin AA, editors. Understanding User Acceptance of IoT Based Healthcare in Jordan: Integration of the TTF and TAM2022. Cham: Springer International Publishing.doi: 10.1007/978-3-031-05258-3_17. (In-text citation: Page 14, Line 264; Page 27, Line 540)*

*10．Zhou C, Qian Y, Kaner J. A study on smart home use intention of elderly consumers based on technology acceptance models. PLOS ONE. 2024; 19(3).doi: 10.1371/journal.pone.0300574. (In-text citation: Page 12, Line 233; Page 17, Line 340; Page 22, Line 443)*

*In addition, we have removed several outdated references and updated them with more recent sources. For details, please refer to the “Reference” section. Newly added references are highlighted in blue, while deleted references are marked in red.*

3. Methodology:

Yes, the method is appropriate. However, it could be improved as follows:

(1) Usually, the research methodology section recommended to include:

a. The type of research you conducted

***Our response:***

*Thank you for your valuable suggestion. We have incorporated the following paragraph into the “Research Methodology” section to clarify the type of research conducted. The specific content is as follows:*

*“This study used structural equation modeling (SEM) for quantitative research. As a multivariate analysis tool, SEM is widely used across various fields because of its ability to comprehensively examine complex relationships between variables [110]. Researchers in this field have adopted SEM for their analyses [10, 46, 50, 61]. As this study aimed to validate the relationships between multiple independent and dependent variables, this method was considered appropriate. SEM includes models based on partial least squares (PLS-SEM) and covariance-based (CB-SEM) [110]. PLS-SEM typically employs Smart PLS software for data analysis, whereas CB-SEM is conducted using Amos” (Page 33, Line 669- Page 34, Line 677).*

b. How you collected and analyzed your data?

***Our response:***

*Thanks very much for your nice comments.* *We provide a detailed explanation of the data collection process in the “Data Collection” section. The specific content is as follows:*

*“Data for this study were collected between March 29, and April 12, 2024 using “WJX”, a well-established and professional online data collection platform in China. In the questionnaire, the researchers explained the basic concepts smart home systems to the respondents and provided detailed instructions to help them understand the study’s context and questions. In total, 550 questionnaires were collected. The researchers cleaned the data and removed responses with excessively short completion times, incomplete answers, and uniform responses. This process yields 475 valid responses” (Page 43, Line 819- Page 44, Line 834).*

*Furthermore, in the “Data Analysis and Results” section, we have included a new description of the data analysis tools and processes, as outlined below:*

*“The data analysis was divided into two parts. The first part involved conducting a descriptive statistical analysis using SPSS 24.0, whereas the second part employed SEM using Smart PLS 4.0. This included an evaluation of both the measurement and structural models. The measurement model analysis focused on verifying the reliability and validity of the variables and indicators, whereas the structural model evaluation was used to test the proposed hypotheses [61]” (Page 44, Line 844- Page 45, Line 849).*

c. Any tools or materials you used in the research?

***Our response:***

*We have added a “Survey instrument” section to clearly outline the data collection tools, as detailed below:*

*“This study used an online questionnaire as a data collection tool based on prior studies in this field [23, 48, 50, 57, 61]. The validity of this method has been well- established, as it is the most widely used survey method in this area of research. The questionnaire was divided into two parts. The first part collected demographic information, and the second part contained measurement items for the research variables, which were adapted from previous studies and appropriately modified to fit the specific context of this research” (Page 34, Line 691- Page 35, Line 701).*

*Furthermore, in the “Data analysis and results” section, we introduce the data collection tools as follows:*

*“The data analysis was divided into two parts. The first part involved conducting a descriptive statistical analysis using SPSS 24.0, whereas the second part employed SEM using Smart PLS 4.0” (Page 44, Line 844- Page 45, Line 846).*

d. How do you mitigate or avoid research biases?

*Thank you for raising this important point. In response, we have added the subsections “Mitigation of positional bias” and “Common method bias (CMB)” to address potential research biases, as detailed below:*

*“****Mitigation of positional bias****：*

*Position bias refers to a phenomenon in which user behavior and selection outcomes are influenced by the positions of items or options on a page or list [122]. To address this issue, a small-scale pilot study was conducted by randomizing the order of the questions and options in the questionnaire to assess potential position bias. The order of the questions and scale items was adjusted based on participant feedback and expert recommendations, and neutral wording was used to minimize language bias. These steps were taken to reduce the impact of position bias on the study results” (Page 46, Line 865- Page 47, Line 872).*

*“****Common method bias (CMB)****：*

*Survey research is often susceptible to common method bias (CMB) [123], which requires careful control. Although previous researchers have used Harman’s single-factor test to detect CMB, the reliability of this method has been questioned [61]. Consequently, scholars recommend using the variance inflation factor (VIF) to detect full collinearity with the commonly accepted threshold of 3.33 [61, 125, 126]. A VIF test was conducted to ensure data accuracy. Table 4 shows that the VIF values in this study from 1.216 to 1.697, all of which are below the specified threshold [124], confirming that there are no CMB issues in the survey data” (Page 47, Line 874- 885).*

***Table 4. Full collinearity.*** *(Page 47, Line 886)*

|  | *BI* | *COM* | *FC* | *PC* | *PEOU* | *PT* | *PU* | *SI* |
| --- | --- | --- | --- | --- | --- | --- | --- | --- |
| *Behavioral intention* |  |  |  |  |  |  |  |  |
| *Compatibility* | *1.617* |  |  |  | *1.216* |  | *1.585* |  |
| *Facilitating conditions* | *1.447* |  |  |  | *1.216* |  | *1.476* |  |
| *Perceived cost* | *1.552* |  |  |  |  |  |  |  |
| *Perceived ease of use* | *1.697* |  |  |  |  |  | *1.673* |  |
| *Perceived trust* | *1.577* |  |  |  |  |  | *1.500* |  |
| *Perceived usefulness* | *1.562* |  |  |  |  |  |  |  |
| *Social influence* |  |  |  |  |  |  | *1.600* |  |

e. Why did you choose these methods?

Please make sure that the “methodology” section includes the above-mentioned elements.

***Our response:***

*Thank you for your valuable advice. We have added a paragraph in the “Research methodology” section to address this point. The details are as follows:*

*“This study used structural equation modeling (SEM) for quantitative research. As a multivariate analysis tool, SEM is widely used across various fields because of its ability to comprehensively examine complex relationships between variables [110]. Researchers in this field have adopted SEM for their analyses [10, 46, 50, 61]. As this study aimed to validate the relationships between multiple independent and dependent variables, this method was considered appropriate” (Page 33, Line 669- Page 34, Line 674).*

*“This study used PLS-SEM for data analysis based on three key considerations. First, compared with CB-SEM, PLS-SEM is more suitable for non-normally distributed data. Because data in this study did not follow a normal distribution, the selection of PLS-SEM ensured the robustness of the analysis and predictive capability of the model [111]. Second, PLS-SEM emphasizes the predictive power and explanatory ability of the model, making it particularly well-suited for models involving multiple variables and complex relationships [112]. As the model in this study involved complex relationships between several variables, PLS-SEM was considered more appropriate. Additionally, PLS-SEM has been widely applied in this field, providing a reference and guidance for our methodological choice [46, 57, 61]” (Page 34, Line 680- Page 34, Line 689).*

(2) Justify the use of the questionnaire

***Our response:***

*We greatly appreciate your suggestions. In the manuscript, we have provided an explanation for the use of questionnaires, as detailed below:*

*“This study used an online questionnaire as a data collection tool based on prior studies in this field [23, 48, 50, 57, 61]. The validity of this method has been well- established, as it is the most widely used survey method in this area of research” (Page 34, Line 692- Page 35, Line 694).*

(3) Why China was selected?

***Our response:***

*Thanks very much for your nice comments. China was chosen for the following reasons. First, in the background part of the “Introduction”, China currently has a serious aging problem, especially in the eastern coastal areas. Secondly, China’s current elderly care method is “aging in place.” The elderly faces the dilemma of physical deterioration and difficulty in living independently. In this regard, the Chinese government actively advocates smart elderly care. Furthermore, through the literature review, we found that there are currently few empirical studies on the acceptance of smart home systems by the elderly based on the Chinese context. Therefore, we selected the eastern coastal area of China for our study. The following is the specific location of the above points in the text:*

*“Since becoming an aging society in 2000, China’s aging process has rapidly accelerated, with the country expected to enter a stage of severe aging by 2035 [1]. According to the ‘Bulletin of the Seventh National Population Census,’ China has the world’s largest elderly population, particularly concentrated in the eastern coastal provinces, such as Shandong, Jiangsu, Guangdong, Hebei, and Zhejiang [4]” (Page 4, Line 68- Page 5, Line 81).*

*“With advancements in the Internet of Things (IoT), information technology, big data, and cloud computing, the Chinese government has been actively promoting smart elderly care [11]” (Page 5, Line 94- 96).*

*“In China, “aging in place” remains the predominant model of elderly care. Statistics indicate that more than half of the elderly population in China lives in empty-nest households [1]. With age, older adults increasingly face physical decline and health challenges [15], leading to a growing need for information technology support [10]” (Page 6, Line 102- 109).*

*“Although existing studies have extensively examined the technical aspects of smart home systems [21, 38, 39], there is a significant lack of empirical research from a user perspective, particularly in the Chinese context” (Page 7, Line 140- 142).*

(4) Justify the selection of this region “the eastern coastal regions of China.

***Our response:***

*Thanks very much for your nice comments. According to China’s latest census, as of 2020, China’s eastern coastal areas have the most severe aging population situation. In addition, as a relatively economically developed area, there are mostly only-child families in this area. China’s current main way of providing care for the elderly is family care, and one-child families are facing even more severe pressure on care for the elderly. As they grow older, their need for technology increases. Therefore, we chose the eastern coastal regions of China. The specific content is as follows：*

*“According to the ‘Bulletin of the Seventh National Population Census,’ China has the world’s largest elderly population, particularly concentrated in the eastern coastal provinces, such as Shandong, Jiangsu, Guangdong, Hebei, and Zhejiang [4]. Fig 1 A presents the top 10 provinces by elderly population, and Fig 1 B illustrates their geographical locations. This trend poses significant challenges across the social, economic, healthcare, and elderly care sectors” (Page 4, Line 79- Page 5, Line 84).*

*“Additionally, the high proportion of one-child families in the eastern region intensifies the burden of elderly care [8]. Age-friendly housing and community planning have become crucial [9]” (Page 5, Line 88- 90).*

*“In China, ‘aging in place’ remains the predominant model of elderly care. Statistics indicate that more than half of the elderly population in China lives in empty-nest households [1]. With age, older adults increasingly face physical decline and health challenges [15], leading to a growing need for information technology support [10]” (Page 6, Line 102- 109).*

(5) “stratified sampling approach” justifies the use of this approach.

***Our response:***

*We appreciate your suggestion and fully agree with it. Accordingly, we have included the rationale for selecting the stratified sampling approach in the “Revised Manuscript with Track Changes,” as detailed below:*

*“Sampling involves selecting a representative subset of units from the overall population [118]. The stratified sampling method divides the population into mutually exclusive strata and then randomly selects samples from each stratum rather than sampling directly from the entire population. This method is advantageous over simple random sampling because it reduces within-stratum heterogeneity, resulting in a more representative sample [119]. In this field, scholars have recommended stratified sampling to enhance representativeness [120]” (Page 40, Line 768- Page 41, Line 774).*

(6) Justify the use of SEM and PLS.

***Our response:***

*Thank you for your valuable suggestions. We have added the reasons for choosing SEM and PLS in the “Revised Manuscript with Track Changes,” as outlined below:*

*“This study used structural equation modeling (SEM) for quantitative research. As a multivariate analysis tool, SEM is widely used across various fields because of its ability to comprehensively examine complex relationships between variables [110]. Researchers in this field have adopted SEM for their analyses [10, 46, 50, 61]. As this study aimed to validate the relationships between multiple independent and dependent variables, this method was considered appropriate” (Page 33, Line 669- Page 34, Line 677).*

*“This study used PLS-SEM for data analysis based on three key considerations. First, compared with CB-SEM, PLS-SEM is more suitable for non-normally distributed data. Because data in this study did not follow a normal distribution, the selection of PLS-SEM ensured the robustness of the analysis and predictive capability of the model [111]. Second, PLS-SEM emphasizes the predictive power and explanatory ability of the model, making it particularly well-suited for models involving multiple variables and complex relationships [112]. As the model in this study involved complex relationships between several variables, PLS-SEM was considered more appropriate. Additionally, PLS-SEM has been widely applied in this field, providing a reference and guidance for our methodological choice [46, 57, 61]” (Page 34, Line 680- 689).*

4. Results

- The results discussion should be linked to the previous studies in the field.

***Our response:***

*Thank you very much for your suggestions. We have made comprehensive adjustments to the “Discussion” section. By carefully examining this section, we discuss the results of this study in the context of previous research. Due to the length, the revised content is not presented here. For full details, please refer to the “Discussion” section in the “Revised Manuscript with Track Changes.” (Page 54, Line 976- Page 63, Line 1164).*

5. General Comments:

(1) At the end of the “introduction section”, the author(s) should include how this manuscript has been organized (the content of each section).

***Our response:***

*We greatly appreciate your suggestion. In response, we have added a paragraph at the end of the “Introduction” section outlining the organization of the article, as detailed below:*

*“The structure of this paper is as follows: The first section introduces the research background, research questions, and objectives. The second section provides a literature review and proposes research hypotheses. The third section details the research methodology, including the processes, methods, and tools used. The fourth section presents the data analysis and results, in which the hypotheses are tested using structural equation modeling. The fifth section discusses the research findings. Finally, the conclusion summarizes the key findings and provides suggestions for future research” (Page 8, Line 149-155).*

(2) Both “theoretical and practical implications” should be improved and linked to the findings of the current study.

***Our response:***

*We appreciate your suggestion and have made extensive revisions accordingly. Based on our findings, we have rewritten the “Theoretical contributions” and “Practical implications” sections. In terms of theoretical contributions, we validated the adaptability of TAM and UTAUT in the context of elderly acceptance of smart home systems, with the inclusion of external variables expanding the existing theoretical model. Additionally, we confirmed the mediating role of perceived usefulness and perceived ease of use between UTAUT variables, external variables, and behavioral intention. The specific details are as follows:*

*“This study examines the key factors influencing older adults’ intentions to use smart home systems, deepens our understanding of their behavioral intentions, and expands the theoretical framework of technology acceptance among the elderly.*

*First, by integrating the TAM, UTAUT, and their extended variables, this study developed a comprehensive model that revealed the complex mechanisms driving older adults’ behavioral intentions toward smart home systems. The findings demonstrate that perceived usefulness, facilitating conditions, compatibility, and perceived cost have significant direct effects on behavioral intention. This validates the applicability of the TAM and UTAUT in this context and underscores the importance of the extended variables (compatibility and perceived cost) in influencing behavioral intention, thereby enriching the existing theoretical framework.*

*In addition, this study explored the mediating effects of perceived ease of use and perceived usefulness. The results demonstrated that facilitating conditions and compatibility indirectly influenced behavioral intention through perceived ease of use, whereas social influence and perceived trust indirectly affected behavioral intention through perceived usefulness. These findings revealed the complex mechanisms underlying the formation of behavioral intentions among the elderly, offering new perspectives for understanding their intentions toward smart home systems and expanding the theoretical model” (Page 68, Line 1264- Page 69, Line 1284).*

*In the “Practical implications” section, we have distinguished between hypotheses with direct and indirect effects on behavioral intention and summarized their practical implications from the perspectives of various stakeholders. Due to the length, the detailed modifications are not included here. For a full overview, please refer to the “Practical implications” section in the “Revised Manuscript with Track Changes” (Page 69, Line 1286- Page 73, Line 1356).*

(3) The author(s) should be clear about which implications reflect the practical perspective and which reflect the theoretical perspective.

***Our response:***

*Thank you for bringing up this important point. As mentioned earlier, we have elaborated on the theoretical contributions by extending the theoretical model and verifying the mediating effects. Additionally, we summarized the impact of various assumptions from the perspectives of different stakeholders and clarified the practical significance of both direct and indirect effects. For detailed revisions, please refer to the “Theoretical contributions” and “Practical implications” sections in the “Revised Manuscript with Track Changes.” The corresponding page numbers for these sections are provided below:*

*- Theoretical Contributions: Page 68, Line 1250- Page 69, Line 1284*

*- Practical Implications: Page 69, Line 1286- Page 73, Line 1356*

6. Quality of Communication:

There is a need for proofreading and professional editing.

*Our response:*

*Thank you for your valuable suggestion. We carefully checked the manuscript and polished it in full through the professional polishing agency “editage.” The polishing certificate is included in the “Appendix” section at the end of the document, please check it out. We sincerely appreciate your comprehensive and detailed feedback on the manuscript.*

Thank you again for your time, effort, and constructive suggestions, which have significantly improved our manuscript. We sincerely hope that our revised manuscript will meet the standards for publication in PLOS ONE. If any further revisions are needed, please feel free to contact us.

Thank you and best regards,

Yuan Wang

School of Housing, Building and Planning, Universiti Sains Malaysia.

E-mail: [yuanwang@student.usm.my](mailto:yuanwang@student.usm.my).

# Appendix:
